# Effectiveness, Efficacy, and Safety of Home-Based Cardiac Rehabilitation Following Valve Replacement or Repair: Protocol for a Systematic Review

**DOI:** 10.2196/86832

**Published:** 2026-08-06

**Authors:** Mohammed Ferid Gharad, Becky Skidmore, George A Wells, Shannon E Kelly

**Affiliations:** 1 School of Epidemiology and Public Health University of Ottawa Ottawa, ON Canada; 2 Cardiovascular Research Methods Centre University of Ottawa Heart Institute Ottawa, ON Canada; 3 Independent Information Specialist Ottawa, ON Canada

**Keywords:** postoperative, cardiac rehabilitation methods, digital rehabilitation, heart valve operation, valvular heart disease, cardiac rehabilitation outcomes, systematic review

## Abstract

**Background:**

Cardiac rehabilitation (CR) is recommended following valve replacement or repair procedures (VRPs), yet participation remains suboptimal among patients undergoing VRPs. Home-based CR (HBCR), including both fully digital and hybrid (mix of HBCR and center-based CR [CBCR]) models, may improve access and uptake. Although prior systematic reviews have examined HBCR in general populations receiving CR, none have specifically considered HBCR among patients who have undergone VRPs—an expanding but under-studied group with distinct rehabilitation needs.

**Objective:**

We aim to perform a systematic review that will evaluate the effectiveness, efficacy, and safety of HBCR compared with CBCR and usual care or no CR in adults following VRPs.

**Methods:**

This systematic review protocol follows guidance from the PRISMA-P (Preferred Reporting Items for Systematic Reviews and Meta-Analyses Protocols) 2015 statement. We will conduct a systematic search in bibliographic databases (MEDLINE, Embase, CENTRAL, CINAHL, and core databases of Web of Science) and ClinicalTrials.gov. Eligible study designs include randomized and nonrandomized controlled trials, controlled observational studies, and single-group cohort or intervention studies. Primary outcomes are major adverse cardiac events, all-cause mortality, and health system resource use. Secondary outcomes are program adherence, quality of life, and patient satisfaction. Two reviewers will independently screen studies for eligibility. One reviewer will perform data extraction using a predefined template, and a second reviewer will independently verify all extracted data for accuracy and completeness. Risk of bias will be assessed using version 2 of the Cochrane risk-of-bias tool for randomized trials, the Risk of Bias in Nonrandomized Studies of Interventions tool, and the National Institutes of Health quality assessment tool for before-and-after (pretest-posttest) studies with no control group. The certainty of evidence will be evaluated using the Grading of Recommendations Assessment, Development, and Evaluation (GRADE) tool. Where appropriate, findings will be synthesized narratively and through meta-analysis.

**Results:**

The review formally began in May 2025 and will be conducted and reported according to the protocol; any changes to this protocol will be documented, updated in the PROSPERO registration, and reported alongside the review findings. As of August 2025, the search identified 398 records, of which 156 (39.2%) were assessed for eligibility, and 21 (5.3%) studies met the inclusion criteria and were retained for data extraction. The results of the review are expected to be published in the first quarter of 2027.

**Conclusions:**

The review aims to focus on clinical-, patient-, and system-important outcomes, which, as a secondary objective, will provide input parameters for planned economic modeling. The findings of this systematic review will inform both practice and policy and provide a foundation for health system–level feasibility assessment, economic modeling, and policy planning related to HBCR in this population.

**Trial Registration:**

PROSPERO CRD420251066703; https://www.crd.york.ac.uk/PROSPERO/view/CRD420251066703

**International Registered Report Identifier (IRRID):**

DERR1-10.2196/86832

## Introduction

Cardiac rehabilitation (CR) is a structured, multicomponent program encompassing exercise training, physical activity promotion, health education, counseling, and cardiovascular risk management designed to assist individuals recovering from cardiovascular events or surgeries, including valve replacement or repair procedures (VRPs) [1]. Traditionally, CR has been delivered in center-based settings such as hospitals, community health service centers, sports centers, or gymnasiums; however, participation rates remain suboptimal due to barriers such as geographic inaccessibility; scheduling conflicts; and limited availability of programs, particularly in rural or underserved areas [2-4]. Furthermore, rising health care costs have constrained the capacity of CR programs, prompting the need for innovative care delivery models [5]. Home-based CR (HBCR) has long been explored as an alternative to traditional, center-based CR (CBCR), offering patients the opportunity to engage in structured exercise and education from the comfort of their own homes or within nonclinical environments such as community centers or local parks [6]. Even though CBCR staff often encourage home-based activities on days when patients are absent from the rehabilitation center, fully independent or “stand-alone” HBCR models have historically been underused [6]. However, the concept of HBCR has gained increasing recognition, with the European guidelines on cardiovascular disease prevention [7] noting that home-based programs (with and without telemonitoring components) hold significant potential for improving participation rates and supporting long-term behavior change [6]. Recent advances in digital health technologies such as remote monitoring, tele-coaching, and e-learning platforms have further expanded the capabilities of HBCR [5]. These innovations, often grouped under the umbrella of digital CR or telerehabilitation, leverage tools such as smartphone apps, web-based platforms, and videoconferencing to deliver evidence-based interventions remotely, offering greater flexibility and accessibility compared to traditional CBCR [5]. These developments position HBCR, including hybrid (mix of HBCR and CBCR) and fully digital approaches, as a promising solution to overcome barriers to participation in CR and extend its reach to patients who might otherwise be excluded from facility-based programs [5].

Among the populations eligible for CR, patients who have undergone VRPs represent a distinct subgroup with unique rehabilitation needs [8]. Referral to CR is generally recommended for patients following heart valve surgery [8,9], although the strength of the existing evidence is still unclear [8].

VRPs are the primary interventions used to treat valvular heart disease [10], which may affect the left side of the heart (aortic and mitral valves), the right side (tricuspid and pulmonary valves), or both sides in rare circumstances [8]. Patients with aortic or mitral valve disease, often caused by either valve narrowing (stenosis) or improper closure (regurgitation), are the most common candidates for VRPs, particularly when symptoms develop or cardiac function begins to decline [10,11]. The different types of VRPs include but are not limited to surgical aortic valve replacement, surgical mitral valve replacement, transcatheter aortic valve replacement, annuloplasty, and chordae repair [12-15].

Despite the presence of robust evidence supporting the effectiveness of CR among post-VRP patients in lowering mortality and morbidity and improving quality of life (QoL) and exercise time [16], research on this topic, particularly the effectiveness of HBCR, is still limited and underexplored. This gap is particularly important for patients who have undergone transcatheter aortic valve replacement, a rapidly growing group with well-documented challenges related to age, frailty, and comorbidity [16,17]. Similar concerns likely apply to patients who have undergone transcatheter mitral valve replacement, although research in this area is still emerging [18]. Despite recommendations [8], participation in CR among post-VRP patients remains low [16]. This raises the need to evaluate whether alternative approaches such as HBCR, which could potentially improve participation, are comparable in effectiveness, efficacy, safety, and economic feasibility to traditional CR (CBCR).

In this review, effectiveness, efficacy, and safety are considered as related but distinct concepts. Efficacy refers to the effect of an intervention under ideal and controlled conditions, whereas effectiveness reflects its impact in routine, real-world clinical settings [19]. Safety relates to the occurrence of adverse clinical events associated with the intervention, using outcomes selected for this review. Although existing systematic reviews have evaluated HBCR in cardiac populations [4,5,20-22], no review to date has specifically examined its use in post-VRP patients, an emerging target group. Accordingly, this review aims to focus on clinical-, patient-, and system-important outcomes. These outcomes will also provide input parameters for planned economic modeling. The findings of this systematic review will inform both practice and policy and provide a foundation for health system–level feasibility assessment, economic modeling, and policy planning related to HBCR in this population.

## Methods

### Study Design

This paper describes a protocol for a systematic review. The protocol was developed a priori and registered with PROSPERO (CRD420251066703).

All research questions will be addressed through a de novo systematic review, which considers published and unpublished studies and studies in progress. This systematic review protocol follows guidance from the PRISMA-P (Preferred Reporting Items for Systematic Reviews and Meta-Analyses Protocols) 2015 statement [23]. A completed PRISMA-P checklist can be found in Multimedia Appendix 1, indicating where each item is addressed within the protocol.

### Review Questions

The primary question is as follows: what is the effectiveness, efficacy, and safety of HBCR compared with CBCR in adults following VRPs? To additionally inform this research question, this review will also explore a number of secondary research questions: (1) How are HBCR programs designed and implemented? What delivery formats are used (eg, synchronous, asynchronous, or hybrid)? What core components are included (eg, type of exercise, education, and psychosocial support)? Who delivers HBCR, and what training is provided? What modes of delivery are used (eg, mobile health technology, telehealth, phone, paper, or a combination of these)? What outcomes are reported in studies of HBCR? (2) How do the effectiveness, efficacy, and safety of HBCR compare with usual care or no CR in post-VRP patients? (3) How do patient or intervention characteristics and contextual factors influence the effectiveness, efficacy, and safety of HBCR compared to CBCR and usual care or no CR (population characteristics [eg, valve type, surgical or nonsurgical VRP, sex, BMI, smoking status, comorbidities, and cardiac history], intervention characteristics [eg, delivery format, modes of delivery, and composition of the program], and contextual factors [eg, geographic setting and health care system differences])?

Answering these review questions will also help us assess how consistent the current evidence base is and to what extent it can inform future economic modeling and system-level decision-making related to HBCR in post-VRP patients.

In this review, the terms “effectiveness,” “efficacy,” and “safety” will be interpreted based on reported clinical, patient-reported, and health system resource use outcomes. Effectiveness refers to the overall impact of HBCR on outcomes such as major adverse cardiac events (MACE), all-cause mortality, health system resource use, and QoL, reflecting its performance in routine or real-world settings [19]. Efficacy refers to the extent to which HBCR demonstrates beneficial effects based on the same outcomes, reflecting its performance under more controlled or structured settings or conditions [19]. Safety will be assessed based on the occurrence of adverse clinical events, including MACE and all-cause mortality.

### Study Eligibility

The eligibility criteria used to address the research questions are summarized in Multimedia Appendix 2. The research questions and eligibility criteria for this review were developed using the population, intervention, comparator, and outcome framework [24]. The eligible population includes adults (18 years or older) following VRPs, and the intervention of interest is HBCR. Comparators include CBCR and usual care or no CR. The outcomes are MACE, all-cause mortality, health system resource use, QoL, program adherence or compliance, and patient satisfaction.

Building on the population, intervention, comparator, and outcome framework, studies will be eligible if they include adults (18 years or older) who have undergone VRPs either surgically or via a transcatheter (nonsurgical) procedure. For our population, all types of VRPs are eligible. The intervention must be HBCR, including digital or virtual, telerehabilitation, or hybrid models that combine home- and center-based components. The intervention must be a CR intervention; in other words, interventions that solely provide postsurgery counseling, follow-up, or support are not eligible. The intervention must at least have an exercise component that is designed for cardiovascular recovery, consistent with the principles of CR [25]. Interventions that focus solely on general physical recovery (eg, strength, mobility, and balance) to restore physical function (ie, support return to daily activities) are not eligible.

Eligible study designs include randomized controlled trials (RCTs), nonrandomized controlled trials (controlled before-and-after studies or interrupted time series), controlled observational studies (ie, prospective, retrospective, or historical cohorts), and single-group cohort or intervention studies. This review is designed to synthesize evidence in an emerging area where high-quality comparative studies remain limited. Accordingly, comparative studies will primarily inform differences between HBCR and CBCR and between HBCR and usual care or no CR, whereas single-group studies and studies comparing different HBCR models without CBCR or usual care or no CR comparators will be included to provide contextual evidence on feasibility and program design.

Primary outcomes, including MACE, all-cause mortality, and health system resource use outcomes, are prioritized because they are widely recognized in the field of CR as some of the most important measures for evaluating the effectiveness, efficacy, and safety of CR interventions. In this review, clinical outcomes such as MACE and all-cause mortality will contribute to the assessment of both effectiveness and safety depending on the direction and magnitude of the observed effects while also informing efficacy assessment by reflecting the benefits observed under study conditions. Health system resource use outcomes will inform effectiveness assessment by reflecting the broader impact of HBCR in practice.

Secondary outcomes such as program adherence, QoL, and patient satisfaction will also be extracted. Program adherence serves as one of the practical indicators of participation and sustained engagement in CR, which is particularly important given the historically low uptake of CR among post-VRP patients [16]. QoL outcomes will add to the clinical findings by reflecting the broader impact of HBCR relative to CBCR on patient well-being and day-to-day functioning [26]. Depending on the study design and context, QoL outcomes may contribute to the assessment of effectiveness and/or efficacy. Patient satisfaction will provide additional insights into how patients perceive and evaluate the overall experience and quality of care they receive [27]. This perspective helps us understand how patients value HBCR relative to CBCR and usual care or no CR.

In addition to their clinical and practical importance, these outcomes were also selected because they represent key input parameters that are important for future economic evaluations of these types of interventions [28]. Clinical outcomes and health system resource use outcomes can be used to inform estimates of intervention effectiveness and cost impact, whereas program adherence, QoL, and patient satisfaction data are essential for modeling real-world effectiveness, utility gains, and long-term value of HBCR within the health care system [28].

No studies will be excluded based on the outcomes reported. In cases in which a study includes a mixed population, we will include it only if the results for post-VRP patients are reported separately as a subgroup. Studies that do not report subgroup data for the eligible population will be excluded.

### Search Strategy and Information Sources

A comprehensive search strategy will be developed in collaboration with an experienced medical information specialist through an iterative process and in consultation with the review team. Prior to execution, another senior information specialist will peer review the MEDLINE strategy using the PRESS (Peer Review of Electronic Search Strategies) checklist [29]. We will search the following bibliographic databases: MEDLINE (Ovid), Embase Classic and Embase (Ovid), CINAHL (EBSCOhost), CENTRAL via Ovid, and the core databases of Web of Science. The search will use a combination of controlled vocabulary (eg, “Heart Valve Diseases/su[surgery],” “Cardiac Rehabilitation,” and “Home Care Services”) and free-text keywords (eg, “TAVI,” “post-repair rehab,” and “telerehab”), with vocabulary and syntax tailored to each database. There will be no language or date restrictions, but where applicable, we will remove animal-only studies, opinion pieces, and pre-2023 conference abstracts. We will download and deduplicate the retrieved records using EndNote (version 9.3.3; Clarivate) and upload these to Covidence (Veritas Health Innovation).

We will screen the reference lists of all included primary studies, as well as systematic reviews of HBCR interventions in broader cardiac populations identified incidentally through the literature search, to identify additional potentially eligible studies. We will also search ClinicalTrials.gov to identify relevant unpublished or ongoing trials. Zotero (version 6.0.37; Corporation for Digital Scholarship) will be the primary reference manager to organize citations and manage references for reporting and manuscript preparation.

We do not plan to use automated alerts or updates during the review process. The final detailed search strategy for all databases can be found in Multimedia Appendix 3. The search strategy is reported in accordance with the PRISMA-S (Preferred Reporting Items for Systematic Reviews and Meta-Analyses literature search extension) guidelines [30].

### Study Screening and Selection

A pilot exercise (using 25 studies) will first be conducted to ensure consistency between the 2 independent reviewers in applying the eligibility criteria. Following the pilot, 2 independent reviewers will screen all titles and abstracts identified through the search against the eligibility criteria. All citations deemed potentially relevant or unclear will undergo full-text screening by the same 2 independent reviewers following another pilot exercise (using 25 studies). Discrepancies at any stage will be resolved through discussion or through consultation with a third reviewer. Screening will be conducted using the Covidence systematic review management application. The complete literature screening and selection process will be documented and presented in a PRISMA (Preferred Reporting Items for Systematic Reviews and Meta-Analyses) flow diagram [23].

### Data Extraction

We will use standardized forms within Covidence to perform data extraction.

All studies included after full-text screening will undergo standardized data extraction using a template developed by the review team, which will be piloted using 5 studies with varying study designs, including RCTs, nonrandomized controlled trials, controlled observational studies, and single-arm studies. One reviewer will extract data from all the included studies, and a second reviewer will independently verify all extracted data for accuracy and completeness. Disagreements will be resolved through discussion or consultation with a third reviewer. Study investigators or corresponding authors of the included studies may be contacted to clarify or obtain missing information where considered essential for the interpretation or synthesis of findings. Any contact, if undertaken, will be limited to a single attempt per study, and the review will otherwise proceed using publicly available data in cases in which responses are not received.

We will extract data from each included study where available on the following elements:

*Study characteristics*, which will include sponsorship source, conflicts of interest, publication type (trial registry record, journal publication, or both), country, author’s contact details (name, institution, email address, and postal address), study start and end date, study status, trial or study registry number, study objective, study design, type of group the study design used (eg, parallel group or crossover), and study setting (single center or multicenter).

*Population characteristics*, which will include inclusion and exclusion criteria, total sample size, number of withdrawals, reason for withdrawals, loss to follow-up, type of valve involved, type of VRP (surgical or transcatheter), and specific name of the procedure (eg, mitral valve repair or surgical aortic valve replacement) extracted at the study level where reported.

*Population characteristics*, which will include the number of participants randomized to or enrolled in each group, age, gender and sex, participants with comorbidities, list of reported comorbidities, participants with additional cardiac history, participants with reoperative VRPs (≥2 procedures), list of reported additional cardiac history, BMI, duration of postoperative hospitalization, and frailty extracted for each study group or arm, where reported, as well as extracting additional variables as appropriate.

Where applicable, we will also extract equity-relevant subgroup information guided by the place of residence; race, ethnicity, culture, or language; occupation; gender and sex; religion; education; socioeconomic status; and social capital (PROGRESS-Plus framework, where “Plus” refers to additional factors that may contribute to disadvantage [eg, age, disability, or other context-specific characteristics]) [31-33].

To ensure comprehensive reporting, we will use the TIDieR (Template for Intervention Description and Replication) checklist [34] to guide the extraction and description of the interventions and comparators. Elements to be extracted for the interventions and comparators include the phase of the CR program; setting; providers or facilitators of the program; training provided to them; theory, rationale, or goal behind the intervention; training provided to participants; core components of the intervention or program; tailoring of the intervention or program; modifications to the intervention or program; modes of delivery; delivery format of the intervention or program; duration of the intervention or program; frequency and duration of each session; session format (group vs individual); monitoring, supervision, and follow-up; and other relevant details. A detailed description of the elements that will be extracted for the interventions and comparators is summarized in Multimedia Appendix 4.

Data extraction will include all primary and secondary outcomes, and any other reported outcomes will be documented.

### Risk-of-Bias Assessment

We will assess the risk of bias at the outcome level using established tools suggested by The Cochrane Collaboration [35] based on study design. Risk-of-bias assessment will be conducted by 2 independent reviewers, with disagreements resolved through discussion or consultation with a third reviewer. Pilot exercises (using 3 studies with varying study designs, including an RCT, controlled observational study, and single-group cohort or intervention study) will be conducted by both reviewers before starting the formal risk-of-bias assessment to ensure consistent interpretation and application of the tools. Narrative summaries of all included studies will highlight their strengths and limitations, and results will be presented and visually summarized using tables or figures.

For RCTs, we will use version 2 of the Cochrane risk-of-bias tool for randomized trials [36]. For all nonrandomized studies evaluating the effects of interventions, including nonrandomized controlled trials and controlled observational studies, we will use the Risk of Bias in Nonrandomized Studies of Interventions tool [37]. For single-group cohort or intervention studies, we will use the National Institutes of Health quality assessment tool for before-and-after (pretest-posttest) studies with no control group [38].

### Meta-Bias

We will assess publication bias in cases in which there are 10 or more included randomized studies using visual tools (eg, funnel plots for RCTs) and statistical tests (eg, Egger regression test or Begg rank correlation test) [35].

### Certainty of Evidence

We will use the Grading of Recommendations Assessment, Development, and Evaluation (GRADE) tool to assess the certainty of evidence across studies for each outcome [39,40]. Evidence from all studies will inform the assessment by a single reviewer. A second independent reviewer will verify the assessments. If needed, any disagreements will be resolved through discussion or consultation with a third reviewer. The GRADE approach evaluates the certainty of a body of evidence based on 5 domains: risk of bias in individual studies, inconsistency, indirectness of evidence, imprecision, and publication bias [41]. The certainty of evidence will be categorized as high, moderate, low, or very low [41]. Findings will be presented in the summary of findings table.

### Synthesis and Data Analyses

Study characteristics and findings will be summarized descriptively and presented in tables. This will include information on study design; population; interventions, comparators, and their characteristics and features; and reported outcomes. Results from all included studies will be summarized narratively. Where appropriate, findings will also be organized and reported according to the relevant research questions and objectives.

Where appropriate and feasible, we will pool the results of the included studies using meta-analyses. Appropriateness will be assessed through reviewer evaluation of the available data for clinical, methodological, and statistical heterogeneity, whereas feasibility will be decided based on the availability of sufficient outcome data. These judgments will be made in consultation with biostatisticians and clinical experts in CR and cardiology. The results from different study designs will not be pooled; instead, separate analyses will be carried out for RCTs, nonrandomized controlled trials, and controlled observational studies. Meta-analyses will not be conducted for studies that do not include a comparison group and studies that compare multiple HBCR arms to each other without our comparators of interest. We will narratively summarize findings from these studies to provide a broader understanding of how HBCR is implemented and its outcomes in post-VRP patients.

Where appropriate and feasible, random-effects meta-analyses will be performed using the Review Manager software (version 5.3 or the most current version; The Cochrane Collaboration). For continuous outcomes, such as QoL or health-related QoL (HRQoL) scores, we will calculate mean differences when the same measurement scale is used. When different measurement scales are used across studies for the same outcome, we will calculate standardized mean differences to enable pooling. For dichotomous outcomes, we will use relative risk or odds ratios depending on study design. Adjusted estimates will be prioritized when available. In cases in which both adjusted and unadjusted results are presented, differences will be explored and discussed. If measures of variability are not reported, we will attempt to impute SEs or SDs using available information. When outcomes are reported as medians and IQRs, we will apply established methods to estimate means and SDs.

Outcomes will be synthesized based on their outcome type and how they are reported. In cases in which multiple studies report similar definitions and use comparable measurement approaches, results will be pooled using relative risks, odds ratios, hazard ratios, or standardized mean differences as appropriate. When pooling is not feasible due to substantial variability in definitions or reporting, findings will be summarized descriptively or narratively.

For all-cause mortality, we will pool results using relative risks or odds ratios when point estimates (eg, proportion of deaths) are reported. If studies report time-to-event outcomes such as survival data (eg, hazard ratios), these will be synthesized separately. For MACE, in addition to synthesizing it as a composite dichotomous outcome [42], individual components (eg, cardiovascular death, myocardial infarction, or stroke) will also be synthesized separately. Each health system resource use outcome (eg, emergency department visits, hospitalizations, intensive care unit admissions, and repeat VRPs) will be synthesized separately. Each program adherence or compliance outcome (eg, completion rates, session attendance, dropout rates, withdrawals due to adverse events, and self-reported adherence) will also be synthesized separately. In cases in which studies report health system resource use or program adherence as composite outcomes, we will also synthesize them as composite outcomes accordingly.

Patient satisfaction outcomes will be synthesized narratively when reported as qualitative feedback. In cases in which quantitative data are available, we will pool results where feasible. QoL or HRQoL scores will be pooled where feasible, prioritizing total scores over subdomain scores.

Findings from individual studies and pooled estimates will be summarized in tables and visualized using forest plots. Additional visual approaches such as harvest plots or albatross plots may also be used [43]. Statistical heterogeneity will be assessed using forest plots, the *I*^2^ statistic, and the Cochran chi-square test. Interpretation of heterogeneity will follow guidance from the *Cochrane Handbook for Systematic Reviews of Interventions* [35]. An *I*^2^ value greater than 75% will be considered indicative of considerable heterogeneity [35].

Clinical characteristics (eg, type of valve involved, surgical or nonsurgical VRP, comorbidities, and cardiac history [including reoperative VRP patients]), intervention characteristics (eg, delivery format and modes of delivery), and contextual factors (eg, geographic setting and health care system differences) will be summarized descriptively across the included studies.

Where feasible, subgroup analyses will explore whether intervention effects differ across these characteristics and factors. Equity-relevant factors will be assessed where feasible using the PROGRESS-Plus framework, including sex and gender, socioeconomic status, race and ethnicity, occupation, religion, education, place of residence (eg, rural vs urban), and disability [31-33]. Additional subgroups may be identified during data extraction or analyses and assessed in an exploratory capacity. All subgroup analyses will be interpreted cautiously, with attention to heterogeneity, risk of bias, and the number of contributing studies.

In cases in which a sufficient number of studies are available, meta-regression analyses may be used to examine the effects of multiple study-level characteristics on intervention outcomes.

Sensitivity analyses may also be conducted to assess the robustness of pooled estimates. These analyses may explore the impact of study quality, year of study, population composition, and other potential sources of bias.

## Results

The review formally began in May 2025 and will be conducted and reported according to the protocol; any changes to this protocol will be documented, updated in the PROSPERO registration, and reported alongside the review findings. Relevant reporting guidelines will be considered when preparing the written report of the systematic review methods and findings (PRISMA [44], PRISMA-Equity [Preferred Reporting Items for Systematic Reviews and Meta-Analyses with a Focus on Health Equity] [45], and the MOOSE [Meta-Analyses of Observational Studies in Epidemiology] checklist [46]).

**Figure 1:**
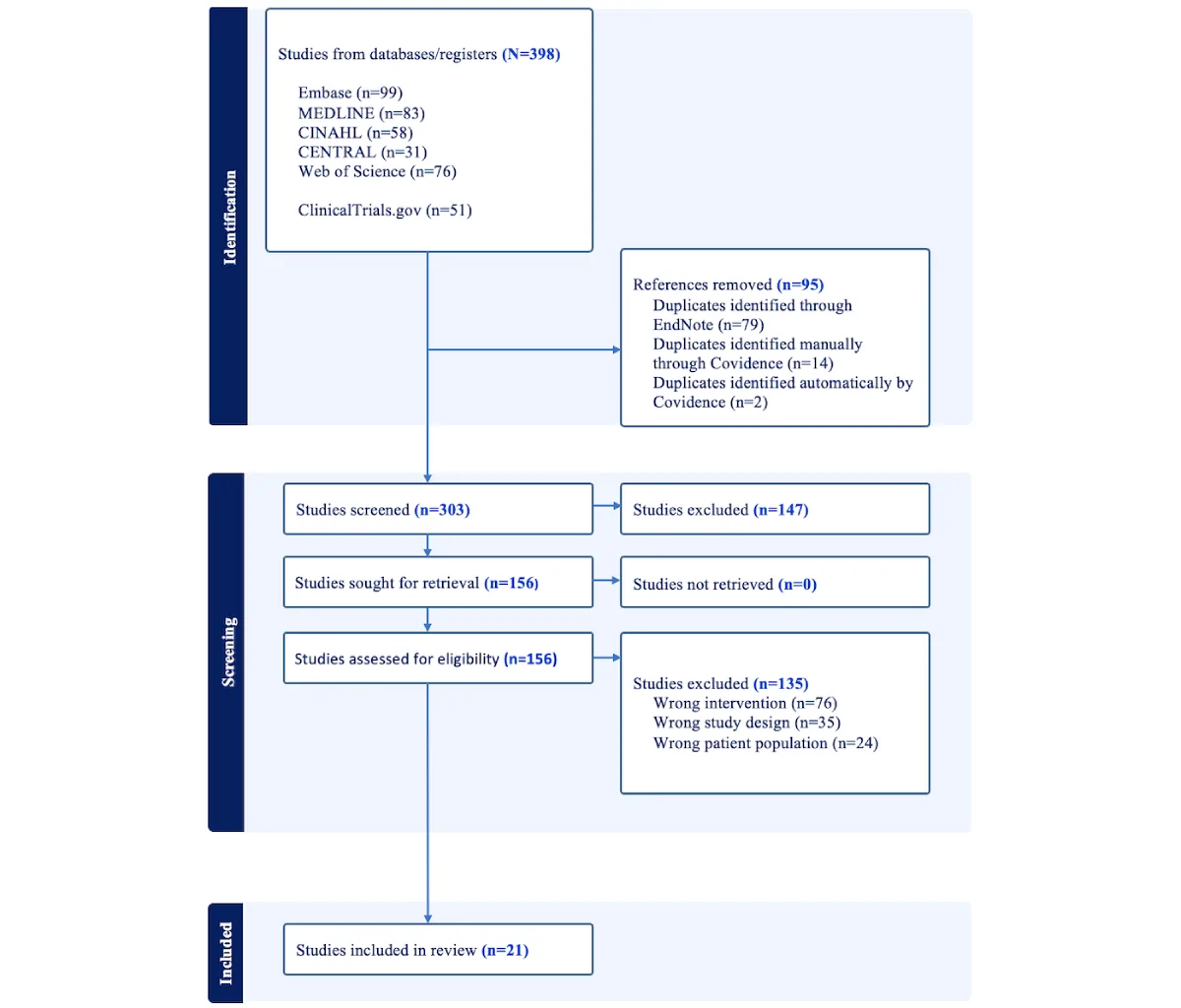
PRISMA (Preferred Reporting Items for Systematic Reviews and Meta-Analyses) 2020 flow diagram.

As of August 2025, our search returned 398 records, of which 156 (39.2%) were assessed for eligibility. Ultimately, 21 studies met the eligibility criteria and were retained for data extraction (Figure 1). Data extraction started in August 2025 and is ongoing. The results of the review are expected to be published in the first quarter of 2027.

## Discussion

### Expected Findings

Given the emerging nature of this clinical area, we anticipate that available evidence will be limited in volume and heterogeneous in terms of study design, intervention characteristics, and outcome reporting. As a result, while comparative findings may be identified, the strength and consistency of evidence across outcomes may be variable, and quantitative synthesis may not be feasible for all outcomes at this stage. As additional studies are published, future updates of this review may be better to support more robust quantitative synthesis, including meta-analysis where appropriate.

In contrast to the limited and heterogeneous evidence anticipated in post-VRP patients, systematic reviews in other cardiac populations have drawn on more established evidence bases. In these populations, HBCR has generally been associated with outcomes comparable to those of CBCR, including clinical outcomes and HRQoL [21,22,47]. Furthermore, a systematic review of digital CR interventions demonstrated that adherence to CR was not inferior compared to CBCR and either QoL was better or no significant difference was observed compared to CBCR groups [48]. Another review has suggested that HBCR has been associated with improved patient satisfaction [49]. However, whether these patterns extend to post-VRP patients remains uncertain. As such, this review will synthesize the available evidence specific to this population, where findings are expected to be more limited and less consistent.

To our knowledge, this review will provide the first systematic synthesis of evidence on the effectiveness, efficacy, and safety of HBCR compared with CBCR and usual care or no CR in post-VRP populations. It will provide a comprehensive summary of delivery models and formats, core components, and outcomes regarding HBCR interventions for post-VRP populations. The findings will be informative for practice and policy (eg, system-level feasibility and informing economic modeling and policy planning) as potential implementation barriers will be identified and evidence gaps will be highlighted.

While our primary focus is on addressing the review questions, we also aim to evaluate how consistent the current evidence base is and the extent to which it can inform economic modeling and system-level decision-making for HBCR in post-VRP patients. To further support this objective, we plan to seek feedback and guidance from a health economist, if feasible.

This patient group has unique needs that make it especially important to evaluate the effectiveness and safety of HBCR. They are typically older adults with greater frailty and multiple comorbidities, factors that may place them at higher clinical risk and influence their response to CR programs [50]. These factors also create additional barriers to participating in CBCR, such as limited mobility and cognitive impairment [51,52], in addition to the universal challenges faced by cardiac patients. Consequently, this population may benefit from alternatives such as HBCR more than other groups.

A key methodological feature of this review protocol is the integration of the TIDieR framework into our data extraction template. Although the TIDieR checklist is widely used in primary intervention studies [34], it is increasingly being used to guide data extraction in systematic reviews, particularly for complex interventions. By incorporating the TIDieR framework into a systematic review of CR interventions, our work offers a reusable methodological template that can improve the consistency and replicability of evidence synthesis in CR and related fields. This ensures that anyone reviewing our protocol can exactly see how intervention and comparator information will be extracted, making the process transparent.

The findings of this review will be disseminated through a peer-reviewed publication, with the aim of informing clinicians, researchers, and health system decision-makers involved in CR and policy planning.

### Limitations

As this is an emerging clinical area, data may be insufficient to quantitatively synthesize the outcomes of interest. This, in turn, may impact our ability to fully address all of our research questions. In cases in which quantitative synthesis is not possible for certain outcomes or no evidence is available, we will transparently report our findings and highlight any gaps in the existing evidence base. By mapping evidence gaps, this review will also identify priorities for future primary studies and highlight areas where economic modeling may be particularly valuable in informing resource allocation.

In addition, the review will rely solely on published data when clarification or additional information cannot be obtained, which may limit the completeness of the evidence synthesis. Any missing or unclear information will be acknowledged when interpreting the findings.

### Conclusions

This protocol outlines a structured approach to synthesizing evidence on the effectiveness, efficacy, and safety of HBCR in post-VRP patients. By integrating clinical, patient-reported, and system-important outcomes, this review aims to provide a comprehensive understanding of HBCR and identify key evidence gaps in this population. The findings are expected to inform future research, policy planning, and economic evaluation in this population.

## Appendix

Multimedia Appendix 1 PRISMA-P 2015 checklist.

Multimedia Appendix 2 Eligibility criteria for the research questions.

Multimedia Appendix 3 Search strategy.

Multimedia Appendix 4 Data extraction template guided by the TIDieR (Template for Intervention Description and Replication) framework for interventions and comparators.

## Abbreviations

CBCR: center-based cardiac rehabilitation
CR: cardiac rehabilitation
GRADE: Grading of Recommendations Assessment, Development, and Evaluation
HBCR: home-based cardiac rehabilitation
HRQoL: health-related quality of life
MACE: major adverse cardiac events
MOOSE: Meta-Analyses of Observational Studies in Epidemiology
PRESS: Peer Review of Electronic Search Strategies
PRISMA: Preferred Reporting Items for Systematic Reviews and Meta-Analyses
PRISMA-Equity: Preferred Reporting Items for Systematic Reviews and Meta-Analyses with a Focus on Health Equity
PRISMA-P: Preferred Reporting Items for Systematic Reviews and Meta-Analyses Protocols
PRISMA-S: Preferred Reporting Items for Systematic Reviews and Meta-Analyses literature search extension
PROGRESS-Plus: place of residence; race, ethnicity, culture, or language; occupation; gender and sex; religion; education; socioeconomic status; and social capital
QoL: quality of life
RCT: randomized controlled trial
TIDieR: Template for Intervention Description and Replication
VRP: valve replacement or repair procedure

## Data Availability

Data sharing is not applicable to this article as no datasets were generated in the process of developing this protocol.
