# Supplementary material for: Effectiveness, Efficacy, and Safety of Home-Based Cardiac Rehabilitation Following Valve Replacement or Repair: Protocol for a Systematic Review
Source: JMIR Res Protoc. 2026 Aug 6;15:e86832. doi: 10.2196/86832 (PMC13494523; doi:10.2196/86832)
Supplement: Supplementary file 2 [file resprot_v15i1e86832_app2.docx]

*Table 2. Eligibility criteria for the research questions.*

*Population*

Adults (≥18 years old) who have undergone valve replacement or repair procedures (VRPs), including surgical or transcatheter (non-surgical) procedures. All types of VRPs are eligible.

Valve replacement procedures, including but not limited to, surgical aortic valve replacement (SAVR), surgical mitral valve replacement (SMVR), transcatheter aortic valve replacement/implantation (TAVR/TAVI), transcatheter mitral valve replacement (TMVR), tricuspid valve replacement, pulmonary valve replacement.

Valve repair procedures, including but not limited to, aortic, mitral, tricuspid, and pulmonary valve repair. Some specific repair techniques that may be performed on some or all these valves include annuloplasty, leaflet repair, and chordae repair.

Exclusions:

- Studies that do not report outcomes separately for post-VRP patients in mixed populations.

*Intervention*

Home-based cardiac rehabilitation (HBCR), delivered in non-clinical settings, including but not limited to the patient’s home, a local park, or a local health club.

This includes:
• In-person HBCR delivered by visiting providers (e.g., nurses).
• Digital or virtual programs (e.g., telehealth, web/portal based).
• Mobile Health (mHealth) technologies (e.g., app-based, SMS-based, programs integrating data from wearables).

• Telephone-based programs.

• Other remote programs (e.g., paper-based).

• Home-based programs that combine multiple modes of delivery (e.g., paper-based + telehealth)

• Hybrid programs (combining home-based and center-based components) – mix of sessions that are conducted at home and sessions that are conducted at CR facilities.

The program must include exercise training, either on its own or as part of a broader cardiac rehabilitation (CR) program that also provides other components like education and psychosocial support. The exercise component must be designed for cardiovascular recovery, consistent with the principles of CR [1].

Exclusions:

- Pre-operative rehabilitation on VRP patients.
- Interventions that focus exclusively on promoting physical activity, weight loss, or a healthy lifestyle through technologies or tools, without offering a structured CR intervention.
- Interventions that focus solely on general physical recovery (e.g., strength, mobility, endurance) to restore physical function, i.e., support return to daily activities.
- Interventions that are primarily center-based, and only include any remote/home-based components as optional, encouragements, or recommendations; rather than as structured/integral parts of the program.

*Comparator*

- Center-based CR (CBCR).
- Usual care/Standard care/no CR: Any routine post-operative care without participation in a structured CR program. This may involve some combination of standard medical follow-up, medication management and adherence, pain management, physical activity recommendations/exercise routines, general lifestyle advice. The specific components may vary depending on local practice and individual patient needs.

*Outcomes*

**1. *Primary Outcomes:***

*• Major adverse cardiac events (MACE)*: A composite outcome that may include, but is not limited to, hospital readmission due to a cardiac event, stroke, myocardial infarction, heart failure, or cardiovascular death, as defined and reported by each included study. Individual components of MACE will be extracted and synthesized separately if reported, regardless of whether the composite MACE outcome is reported.

• *All-cause mortality*, as reported by each included study.

• *Health system resource use*: Including, but not limited to, all-cause hospitalizations (i.e., hospital admissions for any reason), all-cause hospital readmissions, Intensive Care Unit (ICU) admissions, length of hospital/ICU stay, emergency department (ED) visits, and need for repeat valve surgery (which may also be reported as part of a MACE outcome, in some studies), as defined and reported by each included study.
**2. *Secondary Outcomes*:**

• *Program adherence/compliance*: Including, but not limited to, completion rates, session attendance, dropout or withdrawal rates, and self-reported adherence, as defined and reported in each included study.

• Q*uality of life (QoL)* *outcomes* will be included, regardless of whether they are reported as health-related QoL (HRQoL) or general QoL. Established instruments used to measure these outcomes may include the EuroQol-5 Dimensions (EQ-5D), the 36-item Short Form Health Survey (SF-36), the 12-item Short Form Health Survey (SF-12), the World Health Organization Quality of Life – Brief Version (WHOQOL-BREF), and other comparable tools reported in the included studies. Where possible, total scores will be prioritized over subdomain scores.

• *Patient satisfaction*: This may be reported using quantitative measures (e.g., composite satisfaction scores, percentage/proportion of patients satisfied or very satisfied, percentage/proportion of patients who would recommend the program, mean satisfaction score measured on a Likert scale,) or through qualitative feedback on the program.

*Study Design*

Randomised controlled trials, non-randomised controlled trials, quasi-randomised studies, cohort studies (prospective, retrospective, historical), case-control studies, quasi-experimental studies, and single-group cohort or intervention studies that evaluate HBCR in post-VRP patients.

Studies may be from peer-reviewed journal publications, trial registry records, conference abstracts (2023-onwards), protocols, presentations, letters, and academic theses or dissertations. Eligible study protocols and trial registry records will be included, examined for publication bias, and documented or summarized as evidence of “ongoing” research.

Exclusions: case reports, case series, cross-sectional studies, surveys, qualitative studies (e.g., interviews or focus groups), review articles, and opinion-based literature such as editorials, letters to the editor, and commentaries.

*Context/setting*

Any setting or context.

We will explore whether intervention effects are influenced by contextual or setting-related factors. This includes aspects such as urban vs. rural care delivery, and healthcare system differences across regions or countries. Where available, we will extract and synthesize stratified or subgroup results based on place of residence, race/ethnicity/culture/language, occupation, gender/sex, religion, education, socioeconomic status, social capital, and additional context-specific factors that may contribute to disadvantage (PROGRESS-PLUS) [2–4]. We will also note whether any studies report moderating or mediating effects of context/settings on the comparison of HBCR vs. CBCR and the comparison of HBCR vs. usual/standard care.

*Time frame*

No limitations

*Language*

No language limitations

*References*

1.	Brown TM, Pack QR, Aberegg E, Brewer LC, Ford YR, Forman DE, Gathright EC, Khadanga S, Ozemek C, Thomas RJ, on behalf of the American Heart Association Exercise, Cardiac Rehabilitation and Secondary Prevention Committee of the Council on Clinical Cardiology; Council on Cardiovascular and Stroke Nursing; Council on Lifestyle and Cardiometabolic Health; and Council on Quality of Care and Outcomes Research. Core Components of Cardiac Rehabilitation Programs: 2024 Update: A Scientific Statement From the American Heart Association and the American Association of Cardiovascular and Pulmonary Rehabilitation. Circulation American Heart Association; 2024 Oct 29;150(18):e328–e347. PMID:39315436

2.	Whitehead M. The Concepts and Principles of Equity and Health. Int J Health Serv SAGE Publications Inc; 1992 July 1;22(3):429–445. PMID:1644507

3.	O’Neill J, Tabish H, Welch V, Petticrew M, Pottie K, Clarke M, Evans T, Pardo JP, Waters E, White H, Tugwell P. Applying an equity lens to interventions: using PROGRESS ensures consideration of socially stratifying factors to illuminate inequities in health. J Clin Epidemiol Elsevier; 2014 Jan 1;67(1):56–64. PMID:24189091

4.	Hollands GJ, South E, Shemilt I, Oliver S, Thomas J, Sowden AJ. Methods used to conceptualize dimensions of health equity impacts of public health interventions in systematic reviews. J Clin Epidemiol 2024 May 1;169:111312. PMID:38432524
