# Supplementary material for: Effectiveness, Efficacy, and Safety of Home-Based Cardiac Rehabilitation Following Valve Replacement or Repair: Protocol for a Systematic Review
Source: JMIR Res Protoc. 2026 Aug 6;15:e86832. doi: 10.2196/86832 (PMC13494523; doi:10.2196/86832)
Supplement: Supplementary file 3 [file resprot_v15i1e86832_app3.docx]

Cardiac Rehabilitation – Home-Based

Final Strategies

2025 Jun 4

Ovid Multifile

Database: Embase Classic+Embase <1947 to 2025 June 03>, Ovid MEDLINE(R) ALL <1946 to June 03, 2025>, EBM Reviews - Cochrane Central Register of Controlled Trials <April 2025>

Search Strategy:

--------------------------------------------------------------------------------

1	exp Heart Valve Diseases/su [surgery] (114892)

2	exp Heart Valve Prosthesis Implantation/ (145069)

3	(valvo-plast* or valvoplast* or valvo-tom* or valvotom* or valvulo-plast* or valvuloplast* or valvulo-tom* or valvulotom*).tw,kw,kf. (20809)

4	((valve? or valvular) adj5 (implant* or reconstruct* or repair* or replac*)).tw,kw,kf. (194127)

5	((AVI or CVI or HVI or MVI or PVI or TVI or AVR or CVR or HVR or MVR or PVR or TVR) and (valve? or valvular)).tw,kw,kf. (21643)

6	(SAVI or SAVR or SAVI-VIV or SAVI-VinV or SAVR-VIV or SAVR-VinV or VinV-SAVI or VinV-SAVR or ViV-SAVI or ViV-SAVR).tw,kw,kf. (7826)

7	(SMVI or SMVR or SMVI-VIV or SMVI-VinV or SMVR-VIV or SMVR-VinV or VinV-SMVI or VinV-SMVR or ViV-SMVI or ViV-SMVR).tw,kw,kf. (260)

8	(SPVI or SPVR or SPVI-VIV or SPVI-VinV or SPVR-VIV or SPVR-VinV or VinV-SPVI or VinV-SPVR or ViV-SPVI or ViV-SPVR).tw,kw,kf. (425)

9	(STVI or STVR or STVI-VIV or STVI-VinV or STVR-VIV or STVR-VinV or VinV-STVI or VinV-STVR or ViV-STVI or ViV-STVR).tw,kw,kf. (84)

10	(TAVI or TAVR or TAVI-VIV or TAVI-VinV or TAVR-VIV or TAVR-VinV or VinV-TAVI or VinV-TAVR or ViV-TAVI or ViV-TAVR).tw,kw,kf. (45682)

11	(TMVI or TMVR or TMVI-VIV or TMVI-VinV or TMVR-VIV or TMVR-VinV or VinV-TMVI or VinV-TMVR or ViV-TMVI or ViV-TMVR).tw,kw,kf. (2157)

12	(TPVI or TPVR or TPVI-VIV or TPVI-VinV or TPVR-VIV or TPVR-VinV or VinV-TPVI or VinV-TPVR or ViV-TPVI or ViV-TPVR).tw,kw,kf. (878)

13	(TTVI or TTVR or TTVI-VIV or TTVI-VinV or TTVR-VIV or TTVR-VinV or VinV-TTVI or VinV-TTVR or ViV-TTVI or ViV-TTVR).tw,kw,kf. (608)

14	exp Cardiac Valve Annuloplasty/ (12218)

15	(annuloplast* or annulo-plast* or annul* reduc* or annul* repair* or annul* shorten*).tw,kw,kf. (12973)

16	(PTAA or PTMA or PTPA or PTTA).tw,kw,kf. (1521)

17	(chord* adj (reconstruct* or repair* or replac*)).tw,kw,kf. (755)

18	(leaflet adj (reconstruct* or repair* or replac*)).tw,kw,kf. (792)

19	((aorta? or aortic or cardiac or cardio* or heart? or mitral or pulmonar* or tricuspid* or tri-cuspid*) adj2 (valve? or valvular) adj3 (atresia* or incompeten* or insufficien* or prolaps* or regurg* or stenos* or stenotic)).tw,kw,kf. (78431)

20	((aorta? or aortic or mitral or pulmonar* or tricuspid* or tri-cuspid*) adj (incompeten* or insufficien* or prolaps* or regurgitat*)).tw,kw,kf. (129998)

21	((aorta? or aortic or cardiac or cardio* or heart? or mitral or pulmonar* or tricuspid or tri-cuspid) adj2 (valve? or valvular) adj5 (surg* or intervent* or operat* or procedur* or trans-catheter* or transcatheter*)).tw,kw,kf. (124659)

22	or/1-21 [VALVE PROCEDURES, SURGERY] (409055)

23	exp Heart Valve Diseases/rh [rehabilitation] (463)

24	exp Heart Valve Prosthesis Implantation/rh [rehabilitation] (130)

25	Cardiac Rehabilitation/ (24732)

26	((cardiac or cardio* or CVD or heart*) adj3 (recover* or rehab*)).tw,kw,kf. (66897)

27	((post-implant* or postimplant* or post-operat* or postoperat* or post-procedur* or postprocedur* or post-reconstruct* or postreconstruct* or post-repair* or postrepair* or post-replac* or postreplac* or post-surg* or postsurg*) adj3 (recover* or rehab*)).tw,kw,kf. (77321)

28	((post-annuloplast* or post-annulo-plast* or post-annul* reduc* or post-annul* repair* or post-annul* shorten* or postannuloplast* or postannulo-plast* or postannul* reduc* or postannul* repair* or postannul* shorten*) adj3 (recover* or rehab*)).tw,kw,kf. (0)

29	Recovery of Function/ (137594)

30	(recover* adj3 function*).tw,kw,kf. (170455)

31	or/23-30 [CARDIAC/POST-OPERATIVE REHAB] (413793)

32	22 and 31 [VALVE PROCEDURES, SURGERY - REHAB PT 1] (7549)

33	((valve? or valvular) adj5 (implant* or prosthes#s* or prosthetic* or reconstruct* or repair* or replac* or surg* or intervent* or operat* or procedur*) adj3 (recover* or rehab*)).tw,kw,kf. (745)

34	((AVI or CVI or HVI or MVI or PVI or TVI or AVR or CVR or HVR or MVR or PVR or TVR) adj3 (recover* or rehab*)).tw,kw,kf. (399)

35	((SAVI or SAVR or SAVI-VIV or SAVI-VinV or SAVR-VIV or SAVR-VinV or VinV-SAVI or VinV-SAVR or ViV-SAVI or ViV-SAVR) adj3 (recover* or rehab*)).tw,kw,kf. (36)

36	((SMVI or SMVR or SMVI-VIV or SMVI-VinV or SMVR-VIV or SMVR-VinV or VinV-SMVI or VinV-SMVR or ViV-SMVI or ViV-SMVR) adj3 (recover* or rehab*)).tw,kw,kf. (2)

37	((SPVI or SPVR or SPVI-VIV or SPVI-VinV or SPVR-VIV or SPVR-VinV or VinV-SPVI or VinV-SPVR or ViV-SPVI or ViV-SPVR) adj3 (recover* or rehab*)).tw,kw,kf. (4)

38	((STVI or STVR or STVI-VIV or STVI-VinV or STVR-VIV or STVR-VinV or VinV-STVI or VinV-STVR or ViV-STVI or ViV-STVR) adj3 (recover* or rehab*)).tw,kw,kf. (0)

39	((TAVI or TAVR or TAVI-VIV or TAVI-VinV or TAVR-VIV or TAVR-VinV or VinV-TAVI or VinV-TAVR or ViV-TAVI or ViV-TAVR) adj3 (recover* or rehab*)).tw,kw,kf. (336)

40	((TMVI or TMVR or TMVI-VIV or TMVI-VinV or TMVR-VIV or TMVR-VinV or VinV-TMVI or VinV-TMVR or ViV-TMVI or ViV-TMVR) adj3 (recover* or rehab*)).tw,kw,kf. (1)

41	((TPVI or TPVR or TPVI-VIV or TPVI-VinV or TPVR-VIV or TPVR-VinV or VinV-TPVI or VinV-TPVR or ViV-TPVI or ViV-TPVR) adj3 (recover* or rehab*)).tw,kw,kf. (0)

42	((TTVI or TTVR or TTVI-VIV or TTVI-VinV or TTVR-VIV or TTVR-VinV or VinV-TTVI or VinV-TTVR or ViV-TTVI or ViV-TTVR) adj3 (recover* or rehab*)).tw,kw,kf. (1)

43	or/33-42 [VALVE PROCEDURES, SURGERY - REHAB PT 2] (1469)

44	32 or 43 [VALVE PROCEDURES, SURGERY - REHAB - Pts 1-2] (8528)

45	Home Care Services/ (119313)

46	(home-based or homebased or home care or homecare or home health care or home healthcare or home health nurs* or home support* or home treat*).tw,kw,kf. (131077)

47	((care or health care or healthcare or nursing or recover* or rehab* or servic* or support* or treat*) adj3 (domestic or domicil* or home?)).tw,kw,kf. (261198)

48	home setting?.tw,kw,kf. (11476)

49	("at home" or "in home").tw,kw,kf. (241501)

50	((blend* or combo or combination* or hybrid*) adj3 (care or health care or healthcare or nursing or recover* or rehab* or servic* or support* or treat*)).tw,kw,kf. (214221)

51	HBCR.tw,kw,kf. (372)

52	or/45-51 [HOME-BASED] (747402)

53	44 and 52 [VALVE PROCEDURES, SURGERY - HOME-BASED REHAB] (239)

54	Digital Health/ (4770)

55	((digital* or virtual*) adj3 (care or deliver* or health or healthcare or nursing or program* or rehab* or servic* or treat*)).tw,kw,kf. (87933)

56	Internet/ (224818)

57	Therapy, Computer-Assisted/ (13521)

58	(computer* or Internet).ti. (249403)

59	(computer-based or Internet-based or ((computer* or Internet*) adj3 (care or healthcare or nurs* or program* or rehab* or service?))).tw,kw,kf. (140465)

60	Smartphone/ (50425)

61	(smart phone? or smartphone?).tw,kw,kf. (86801)

62	Mobile Applications/ (46063)

63	(app-based or application-based).tw,kw,kf. (10912)

64	((computer* or internet* or i-phon* or iphon* or mobile or online or portable or smart phone* or smartphone* or software or web) adj5 (app or apps or application? or rehab*)).tw,kw,kf. (144038)

65	Telemedicine/ (103733)

66	(e-health or ehealth or e-med* or emed* or remote med* or tele care or telecare or tele-med* or telemed* or virtual care or virtual med*).tw,kw,kf. (116912)

67	Telenursing/ (846)

68	(e-nurs* or enurs* or remote nurs* or tele nurs* or telenurs* or virtual nurs*).tw,kw,kf. (2069)

69	Telerehabilitation/ (6109)

70	(e-rehab* or remote rehab* or tele-rehab* or telerehab* or virtual rehab*).tw,kw,kf. (10231)

71	((care or healthcare or nurs* or program* or rehab* or service?) adj3 (digital* or mobile or remote* or tele* or virtual*)).tw,kw,kf. (129561)

72	or/54-71 [DIGITAL/VIRTUAL PROGRAMS] (954130)

73	44 and 72 [VALVE PROCEDURES, SURGERY - DIGITAL/VIRTUAL PROGRAMS] (118)

74	Self Care/ (132658)

75	Self-Management/ (98050)

76	((personal or self) adj3 (care or manag*)).tw,kw,kf. (216629)

77	or/74-76 [SELF CARE/MANAGEMENT] (266853)

78	44 and 77 [VALVE PROCEDURES, SURGERY - SELF CARE/MANAGEMENT] (61)

79	53 or 73 or 78 [VALVE PROCEDURES, SURGERY - HOME-BASED REHAB, DIGITAL/VIRTUAL, SELF CARE] (348)

80	exp Animals/ not Humans/ (12242459)

81	79 not 80 [ANIMAL-ONLY REMOVED] (346)

82	(comment or editorial or news or newspaper article or (letter not randomized controlled trial)).pt. (4773227)

83	81 not 82 [OPINION PIECES REMOVED] (341)

84	83 use medall [MEDLINE RECORDS] (84)

85	exp valvular heart disease/su [Surgery] (114892)

86	exp heart valve replacement/ (103372)

87	(valvo-plast* or valvoplast* or valvo-tom* or valvotom* or valvulo-plast* or valvuloplast* or valvulo-tom* or valvulotom*).tw,kw,kf. (20809)

88	((valve? or valvular) adj5 (implant* or reconstruct* or repair* or replac*)).tw,kw,kf. (194127)

89	((AVI or CVI or HVI or MVI or PVI or TVI or AVR or CVR or HVR or MVR or PVR or TVR) and (valve? or valvular)).tw,kw,kf. (21643)

90	(SAVI or SAVR or SAVI-VIV or SAVI-VinV or SAVR-VIV or SAVR-VinV or VinV-SAVI or VinV-SAVR or ViV-SAVI or ViV-SAVR).tw,kw,kf. (7826)

91	(SMVI or SMVR or SMVI-VIV or SMVI-VinV or SMVR-VIV or SMVR-VinV or VinV-SMVI or VinV-SMVR or ViV-SMVI or ViV-SMVR).tw,kw,kf. (260)

92	(SPVI or SPVR or SPVI-VIV or SPVI-VinV or SPVR-VIV or SPVR-VinV or VinV-SPVI or VinV-SPVR or ViV-SPVI or ViV-SPVR).tw,kw,kf. (425)

93	(STVI or STVR or STVI-VIV or STVI-VinV or STVR-VIV or STVR-VinV or VinV-STVI or VinV-STVR or ViV-STVI or ViV-STVR).tw,kw,kf. (84)

94	(TAVI or TAVR or TAVI-VIV or TAVI-VinV or TAVR-VIV or TAVR-VinV or VinV-TAVI or VinV-TAVR or ViV-TAVI or ViV-TAVR).tw,kw,kf. (45682)

95	(TMVI or TMVR or TMVI-VIV or TMVI-VinV or TMVR-VIV or TMVR-VinV or VinV-TMVI or VinV-TMVR or ViV-TMVI or ViV-TMVR).tw,kw,kf. (2157)

96	(TPVI or TPVR or TPVI-VIV or TPVI-VinV or TPVR-VIV or TPVR-VinV or VinV-TPVI or VinV-TPVR or ViV-TPVI or ViV-TPVR).tw,kw,kf. (878)

97	(TTVI or TTVR or TTVI-VIV or TTVI-VinV or TTVR-VIV or TTVR-VinV or VinV-TTVI or VinV-TTVR or ViV-TTVI or ViV-TTVR).tw,kw,kf. (608)

98	exp annuloplasty/ (9089)

99	(annuloplast* or annulo-plast* or annul* reduc* or annul* repair* or annul* shorten*).tw,kw,kf. (12973)

100	(PTAA or PTMA or PTPA or PTTA).tw,kw,kf. (1521)

101	(chord* adj (reconstruct* or repair* or replac*)).tw,kw,kf. (755)

102	(leaflet adj (reconstruct* or repair* or replac*)).tw,kw,kf. (792)

103	((aorta? or aortic or cardiac or cardio* or heart? or mitral or pulmonar* or tricuspid* or tri-cuspid*) adj2 (valve? or valvular) adj3 (atresia* or incompeten* or insufficien* or prolaps* or regurg* or stenos* or stenotic)).tw,kw,kf. (78431)

104	((aorta? or aortic or mitral or pulmonar* or tricuspid* or tri-cuspid*) adj (incompeten* or insufficien* or prolaps* or regurgitat*)).tw,kw,kf. (129998)

105	((aorta? or aortic or cardiac or cardio* or heart? or mitral or pulmonar* or tricuspid or tri-cuspid) adj2 (valve? or valvular) adj5 (surg* or intervent* or operat* or procedur* or trans-catheter* or transcatheter*)).tw,kw,kf. (124659)

106	or/85-105 [VALVE PROCEDURES, SURGERY] (405825)

107	exp valvular heart disease/rh [rehabilitation] (463)

108	exp heart valve replacement/rh [rehabilitation] (16)

109	heart rehabilitation/ (19663)

110	((cardiac or cardio* or CVD or heart*) adj3 (recover* or rehab*)).tw,kw,kf. (66897)

111	((post-implant* or postimplant* or post-operat* or postoperat* or post-procedur* or postprocedur* or post-reconstruct* or postreconstruct* or post-repair* or postrepair* or post-replac* or postreplac* or post-surg* or postsurg*) adj3 (recover* or rehab*)).tw,kw,kf. (77321)

112	((post-annuloplast* or post-annulo-plast* or post-annul* reduc* or post-annul* repair* or post-annul* shorten* or postannuloplast* or postannulo-plast* or postannul* reduc* or postannul* repair* or postannul* shorten*) adj3 (recover* or rehab*)).tw,kw,kf. (0)

113	convalescence/ (71485)

114	(recover* adj3 function*).tw,kw,kf. (170455)

115	or/107-114 [CARDIAC/POST-OPERATIVE REHAB] (362438)

116	106 and 115 [VALVE REPLACEMENT SURGERY - REHAB PT 1] (5810)

117	((valve? or valvular) adj5 (implant* or repair* or replac* or surg* or intervent* or operat* or procedur*) adj3 (recover* or rehab*)).tw,kw,kf. (691)

118	((AVI or CVI or HVI or MVI or PVI or TVI or AVR or CVR or HVR or MVR or PVR or TVR) adj3 (recover* or rehab*)).tw,kw,kf. (399)

119	((SAVI or SAVR or SAVI-VIV or SAVI-VinV or SAVR-VIV or SAVR-VinV or VinV-SAVI or VinV-SAVR or ViV-SAVI or ViV-SAVR) adj3 (recover* or rehab*)).tw,kw,kf. (36)

120	((SMVI or SMVR or SMVI-VIV or SMVI-VinV or SMVR-VIV or SMVR-VinV or VinV-SMVI or VinV-SMVR or ViV-SMVI or ViV-SMVR) adj3 (recover* or rehab*)).tw,kw,kf. (2)

121	((SPVI or SPVR or SPVI-VIV or SPVI-VinV or SPVR-VIV or SPVR-VinV or VinV-SPVI or VinV-SPVR or ViV-SPVI or ViV-SPVR) adj3 (recover* or rehab*)).tw,kw,kf. (4)

122	((STVI or STVR or STVI-VIV or STVI-VinV or STVR-VIV or STVR-VinV or VinV-STVI or VinV-STVR or ViV-STVI or ViV-STVR) adj3 (recover* or rehab*)).tw,kw,kf. (0)

123	((TAVI or TAVR or TAVI-VIV or TAVI-VinV or TAVR-VIV or TAVR-VinV or VinV-TAVI or VinV-TAVR or ViV-TAVI or ViV-TAVR) adj3 (recover* or rehab*)).tw,kw,kf. (336)

124	((TMVI or TMVR or TMVI-VIV or TMVI-VinV or TMVR-VIV or TMVR-VinV or VinV-TMVI or VinV-TMVR or ViV-TMVI or ViV-TMVR) adj3 (recover* or rehab*)).tw,kw,kf. (1)

125	((TPVI or TPVR or TPVI-VIV or TPVI-VinV or TPVR-VIV or TPVR-VinV or VinV-TPVI or VinV-TPVR or ViV-TPVI or ViV-TPVR) adj3 (recover* or rehab*)).tw,kw,kf. (0)

126	((TTVI or TTVR or TTVI-VIV or TTVI-VinV or TTVR-VIV or TTVR-VinV or VinV-TTVI or VinV-TTVR or ViV-TTVI or ViV-TTVR) adj3 (recover* or rehab*)).tw,kw,kf. (1)

127	or/117-126 [VALVE PROCEDURES, SURGERY - REHAB PT 2] (1415)

128	116 or 127 [VALVE PROCEDURES, SURGERY - REHAB - Pts 1-2] (6762)

129	home care/ (119313)

130	home rehabilitation/ (1513)

131	(home-based or homebased or home care or homecare or home health care or home healthcare or home health nurs* or home support* or home treat*).tw,kw,kf. (131077)

132	((care or health care or healthcare or nursing or recover* or rehab* or servic* or support* or treat*) adj3 (domestic or domicil* or home?)).tw,kw,kf. (261198)

133	home setting?.tw,kw,kf. (11476)

134	("at home" or "in home").tw,kw,kf. (241501)

135	((blend* or combo or combination* or hybrid*) adj3 (care or health care or healthcare or nursing or recover* or rehab* or servic* or support* or treat*)).tw,kw,kf. (214221)

136	HBCR.tw,kw,kf. (372)

137	or/129-136 [HOME-BASED] (747782)

138	128 and 137 [HOME-BASED - VALVE REPLACEMENT SURGERY REHAB] (236)

139	exp digital health/ (5501)

140	((digital* or virtual*) adj3 (care or deliver* or health or healthcare or nursing or program* or rehab* or servic* or treat*)).tw,kw,kf. (87933)

141	internet/ (224818)

142	computer assisted therapy/ (13521)

143	(computer* or Internet).ti. (249403)

144	(computer-based or Internet-based or ((computer* or Internet*) adj3 (care or healthcare or nurs* or program* or rehab* or service?))).tw,kw,kf. (140465)

145	exp mobile phone/ (91828)

146	(smart phone? or smartphone?).tw,kw,kf. (86801)

147	exp mobile application/ (51759)

148	(app-based or application-based).tw,kw,kf. (10912)

149	((computer* or internet* or i-phon* or iphon* or mobile or online or portable or smart phone* or smartphone* or software or web) adj5 (app or apps or application? or rehab*)).tw,kw,kf. (144038)

150	telemedicine/ (103733)

151	(e-health or ehealth or e-med* or emed* or remote med* or tele care or telecare or tele-med* or telemed* or virtual care or virtual med*).tw,kw,kf. (116912)

152	telenursing/ (846)

153	(e-nurs* or enurs* or remote nurs* or tele nurs* or telenurs* or virtual nurs*).tw,kw,kf. (2069)

154	telerehabilitation/ (6109)

155	(e-rehab* or remote rehab* or tele-rehab* or telerehab* or virtual rehab*).tw,kw,kf. (10231)

156	((care or healthcare or nurs* or program* or rehab* or service?) adj3 (digital* or mobile or remote* or tele* or virtual*)).tw,kw,kf. (129561)

157	or/139-156 [DIGITAL/VIRTUAL PROGRAMS] (981149)

158	128 and 157 [DIGITAL/VIRTUAL PROGRAMS - VALVE REPLACEMENT SURGERY] (116)

159	self care/ (132658)

160	((personal or self) adj3 (care or manag*)).tw,kw,kf. (216629)

161	or/159-160 [SELF CARE/MANAGEMENT] (266180)

162	128 and 161 [SELF CARE/MANAGEMENT - VALVE REPLACEMENT SURGERY REHAB] (51)

163	138 or 158 or 162 [HOME-BASED, DIGITAL/VIRTUAL, SELF CARE - VALVE REPLACEMENT SURGERY REHAB] (334)

164	(exp animal/ or exp animal model/ or exp animal experiment/ or nonhuman/ or exp vertebrate/) not (exp human/ or exp human experiment/) (14095166)

165	163 not 164 [ANIMAL-ONLY REMOVED] (331)

166	editorial.pt. (1568659)

167	letter.pt. not randomized controlled trial/ (2659988)

168	165 not (166 or 167) [OPINION PIECES REMOVED] (327)

169	conference abstract.pt. (5486830)

170	168 not 169 (255)

171	168 and 169 (72)

172	limit 171 to yr="2023-current" (13)

173	170 or 172 [MOST RECENT 2 YRS CONFERENCE ABSTRACTS RETAINED] (268)

174	173 use emczd [EMBASE RECORDS] (152)

175	exp Heart Valve Diseases/su [surgery] (114892)

176	exp Heart Valve Prosthesis Implantation/ (145069)

177	(valvo-plast* or valvoplast* or valvo-tom* or valvotom* or valvulo-plast* or valvuloplast* or valvulo-tom* or valvulotom*).ti,ab,kw. (20474)

178	((valve? or valvular) adj5 (implant* or reconstruct* or repair* or replac*)).ti,ab,kw. (188751)

179	((AVI or CVI or HVI or MVI or PVI or TVI or AVR or CVR or HVR or MVR or PVR or TVR) and (valve? or valvular)).ti,ab,kw. (21246)

180	(SAVI or SAVR or SAVI-VIV or SAVI-VinV or SAVR-VIV or SAVR-VinV or VinV-SAVI or VinV-SAVR or ViV-SAVI or ViV-SAVR).ti,ab,kw. (7697)

181	(SMVI or SMVR or SMVI-VIV or SMVI-VinV or SMVR-VIV or SMVR-VinV or VinV-SMVI or VinV-SMVR or ViV-SMVI or ViV-SMVR).ti,ab,kw. (259)

182	(SPVI or SPVR or SPVI-VIV or SPVI-VinV or SPVR-VIV or SPVR-VinV or VinV-SPVI or VinV-SPVR or ViV-SPVI or ViV-SPVR).ti,ab,kw. (425)

183	(STVI or STVR or STVI-VIV or STVI-VinV or STVR-VIV or STVR-VinV or VinV-STVI or VinV-STVR or ViV-STVI or ViV-STVR).ti,ab,kw. (84)

184	(TAVI or TAVR or TAVI-VIV or TAVI-VinV or TAVR-VIV or TAVR-VinV or VinV-TAVI or VinV-TAVR or ViV-TAVI or ViV-TAVR).ti,ab,kw. (45227)

185	(TMVI or TMVR or TMVI-VIV or TMVI-VinV or TMVR-VIV or TMVR-VinV or VinV-TMVI or VinV-TMVR or ViV-TMVI or ViV-TMVR).ti,ab,kw. (2091)

186	(TPVI or TPVR or TPVI-VIV or TPVI-VinV or TPVR-VIV or TPVR-VinV or VinV-TPVI or VinV-TPVR or ViV-TPVI or ViV-TPVR).ti,ab,kw. (863)

187	(TTVI or TTVR or TTVI-VIV or TTVI-VinV or TTVR-VIV or TTVR-VinV or VinV-TTVI or VinV-TTVR or ViV-TTVI or ViV-TTVR).ti,ab,kw. (598)

188	exp Cardiac Valve Annuloplasty/ (12218)

189	(annuloplast* or annulo-plast* or annul* reduc* or annul* repair* or annul* shorten*).ti,ab,kw. (12604)

190	(PTAA or PTMA or PTPA or PTTA).ti,ab,kw. (1512)

191	(chord* adj (reconstruct* or repair* or replac*)).ti,ab,kw. (736)

192	(leaflet adj (reconstruct* or repair* or replac*)).ti,ab,kw. (759)

193	((aorta? or aortic or cardiac or cardio* or heart? or mitral or pulmonar* or tricuspid* or tri-cuspid*) adj2 (valve? or valvular) adj3 (atresia* or incompeten* or insufficien* or prolaps* or regurg* or stenos* or stenotic)).ti,ab,kw. (68775)

194	((aorta? or aortic or mitral or pulmonar* or tricuspid* or tri-cuspid*) adj (incompeten* or insufficien* or prolaps* or regurgitat*)).ti,ab,kw. (126132)

195	((aorta? or aortic or cardiac or cardio* or heart? or mitral or pulmonar* or tricuspid or tri-cuspid) adj2 (valve? or valvular) adj5 (surg* or intervent* or operat* or procedur* or trans-catheter* or transcatheter*)).ti,ab,kw. (119268)

196	or/175-195 [VALVE PROCEDURES, SURGERY] (402368)

197	exp Heart Valve Diseases/rh [rehabilitation] (463)

198	exp Heart Valve Prosthesis Implantation/rh [rehabilitation] (130)

199	Cardiac Rehabilitation/ (24732)

200	((cardiac or cardio* or CVD or heart*) adj3 (recover* or rehab*)).ti,ab,kw. (65477)

201	((post-implant* or postimplant* or post-operat* or postoperat* or post-procedur* or postprocedur* or post-reconstruct* or postreconstruct* or post-repair* or postrepair* or post-replac* or postreplac* or post-surg* or postsurg*) adj3 (recover* or rehab*)).ti,ab,kw. (76508)

202	((post-annuloplast* or post-annulo-plast* or post-annul* reduc* or post-annul* repair* or post-annul* shorten* or postannuloplast* or postannulo-plast* or postannul* reduc* or postannul* repair* or postannul* shorten*) adj3 (recover* or rehab*)).ti,ab,kw. (0)

203	Recovery of Function/ (137594)

204	(recover* adj3 function*).ti,ab,kw. (167737)

205	or/197-204 [CARDIAC/POST-OPERATIVE REHAB] (410113)

206	196 and 205 [VALVE PROCEDURES, SURGERY - REHAB PT 1] (7458)

207	((valve? or valvular) adj5 (implant* or prosthes#s* or prosthetic* or reconstruct* or repair* or replac* or surg* or intervent* or operat* or procedur*) adj3 (recover* or rehab*)).ti,ab,kw. (742)

208	((AVI or CVI or HVI or MVI or PVI or TVI or AVR or CVR or HVR or MVR or PVR or TVR) adj3 (recover* or rehab*)).ti,ab,kw. (399)

209	((SAVI or SAVR or SAVI-VIV or SAVI-VinV or SAVR-VIV or SAVR-VinV or VinV-SAVI or VinV-SAVR or ViV-SAVI or ViV-SAVR) adj3 (recover* or rehab*)).ti,ab,kw. (36)

210	((SMVI or SMVR or SMVI-VIV or SMVI-VinV or SMVR-VIV or SMVR-VinV or VinV-SMVI or VinV-SMVR or ViV-SMVI or ViV-SMVR) adj3 (recover* or rehab*)).ti,ab,kw. (2)

211	((SPVI or SPVR or SPVI-VIV or SPVI-VinV or SPVR-VIV or SPVR-VinV or VinV-SPVI or VinV-SPVR or ViV-SPVI or ViV-SPVR) adj3 (recover* or rehab*)).ti,ab,kw. (4)

212	((STVI or STVR or STVI-VIV or STVI-VinV or STVR-VIV or STVR-VinV or VinV-STVI or VinV-STVR or ViV-STVI or ViV-STVR) adj3 (recover* or rehab*)).ti,ab,kw. (0)

213	((TAVI or TAVR or TAVI-VIV or TAVI-VinV or TAVR-VIV or TAVR-VinV or VinV-TAVI or VinV-TAVR or ViV-TAVI or ViV-TAVR) adj3 (recover* or rehab*)).ti,ab,kw. (335)

214	((TMVI or TMVR or TMVI-VIV or TMVI-VinV or TMVR-VIV or TMVR-VinV or VinV-TMVI or VinV-TMVR or ViV-TMVI or ViV-TMVR) adj3 (recover* or rehab*)).ti,ab,kw. (1)

215	((TPVI or TPVR or TPVI-VIV or TPVI-VinV or TPVR-VIV or TPVR-VinV or VinV-TPVI or VinV-TPVR or ViV-TPVI or ViV-TPVR) adj3 (recover* or rehab*)).ti,ab,kw. (0)

216	((TTVI or TTVR or TTVI-VIV or TTVI-VinV or TTVR-VIV or TTVR-VinV or VinV-TTVI or VinV-TTVR or ViV-TTVI or ViV-TTVR) adj3 (recover* or rehab*)).ti,ab,kw. (1)

217	or/207-216 [VALVE PROCEDURES, SURGERY - REHAB PT 2] (1465)

218	206 or 217 [VALVE PROCEDURES, SURGERY - REHAB - Pts 1-2] (8441)

219	Home Care Services/ (119313)

220	(home-based or homebased or home care or homecare or home health care or home healthcare or home health nurs* or home support* or home treat*).ti,ab,kw. (128999)

221	((care or health care or healthcare or nursing or recover* or rehab* or servic* or support* or treat*) adj3 (domestic or domicil* or home?)).ti,ab,kw. (254754)

222	home setting?.ti,ab,kw. (11472)

223	("at home" or "in home").ti,ab,kw. (241029)

224	((blend* or combo or combination* or hybrid*) adj3 (care or health care or healthcare or nursing or recover* or rehab* or servic* or support* or treat*)).ti,ab,kw. (212902)

225	HBCR.ti,ab,kw. (368)

226	or/219-225 [HOME-BASED] (741535)

227	218 and 226 [VALVE PROCEDURES, SURGERY - HOME-BASED REHAB] (237)

228	Digital Health/ (4770)

229	((digital* or virtual*) adj3 (care or deliver* or health or healthcare or nursing or program* or rehab* or servic* or treat*)).ti,ab,kw. (79124)

230	Internet/ (224818)

231	Therapy, Computer-Assisted/ (13521)

232	(computer* or Internet).ti. (249403)

233	(computer-based or Internet-based or ((computer* or Internet*) adj3 (care or healthcare or nurs* or program* or rehab* or service?))).ti,ab,kw. (138329)

234	Smartphone/ (50425)

235	(smart phone? or smartphone?).ti,ab,kw. (85937)

236	Mobile Applications/ (46063)

237	(app-based or application-based).ti,ab,kw. (10842)

238	((computer* or internet* or i-phon* or iphon* or mobile or online or portable or smart phone* or smartphone* or software or web) adj5 (app or apps or application? or rehab*)).ti,ab,kw. (140024)

239	Telemedicine/ (103733)

240	(e-health or ehealth or e-med* or emed* or remote med* or tele care or telecare or tele-med* or telemed* or virtual care or virtual med*).ti,ab,kw. (116007)

241	Telenursing/ (846)

242	(e-nurs* or enurs* or remote nurs* or tele nurs* or telenurs* or virtual nurs*).ti,ab,kw. (2065)

243	Telerehabilitation/ (6109)

244	(e-rehab* or remote rehab* or tele-rehab* or telerehab* or virtual rehab*).ti,ab,kw. (10212)

245	((care or healthcare or nurs* or program* or rehab* or service?) adj3 (digital* or mobile or remote* or tele* or virtual*)).ti,ab,kw. (127704)

246	or/228-245 [DIGITAL/VIRTUAL PROGRAMS] (946709)

247	218 and 246 [DIGITAL/VIRTUAL PROGRAMS - VALVE REPLACEMENT SURGERY] (115)

248	Self Care/ (132658)

249	Self-Management/ (98050)

250	((personal or self) adj3 (care or manag*)).ti,ab,kw. (210578)

251	or/248-250 [SELF CARE/MANAGEMENT] (262247)

252	218 and 251 [VALVE PROCEDURES, SURGERY - SELF CARE/MANAGEMENT] (55)

253	227 or 247 or 252 [HOME-BASED, DIGITAL/VIRTUAL, SELF CARE - VALVE REPLACEMENT SURGERY REHAB] (342)

254	conference proceeding.pt. (256112)

255	253 not 254 (339)

256	253 and 254 (3)

257	limit 256 to yr="2023-current" (1)

258	255 or 257 [MOST RECENT 2 YRS CONFERENCE ABSTRACTS RETAINED] (340)

259	258 use cctr [CENTRAL RECORDS] (48)

260	84 or 174 or 259 [ALL DATABASES] (284)

261	remove duplicates from 260 (213) [**TOTAL UNIQUE RECORDS**]

262	261 use medall [MEDLINE UNIQUE RECORDS] (83)

263	261 use emczd [EMBASE UNIQUE RECORDS] (99)

264	261 use cctr [CENTRAL UNIQUE RECORDS] (31)

***************************

CINAHL

| # | Query | Limiters/Expanders | Last Run Via | Results |
| --- | --- | --- | --- | --- |
| S83 | PT (S81 NOT S82) | Expanders - Apply related words; Apply equivalent subjects  Search modes - Proximity | Interface - EBSCOhost Research Databases  Search Screen - Advanced Search  Database - CINAHL | 58 |
| S82 | PT editorial | Expanders - Apply related words; Apply equivalent subjects  Search modes - Proximity | Interface - EBSCOhost Research Databases  Search Screen - Advanced Search  Database - CINAHL | 286,258 |
| S81 | S54 OR S75 OR S80 | Expanders - Apply related words; Apply equivalent subjects  Search modes - Proximity | Interface - EBSCOhost Research Databases  Search Screen - Advanced Search  Database - CINAHL | 58 |
| S80 | S44 AND S79 | Expanders - Apply related words; Apply equivalent subjects  Search modes - Proximity | Interface - EBSCOhost Research Databases  Search Screen - Advanced Search  Database - CINAHL | 15 |
| S79 | S76 OR S77 OR S78 | Expanders - Apply related words; Apply equivalent subjects  Search modes - Proximity | Interface - EBSCOhost Research Databases  Search Screen - Advanced Search  Database - CINAHL | 81,872 |
| S78 | XB ((personal or self) N3 (care or manag*)) | Expanders - Apply related words; Apply equivalent subjects  Search modes - Proximity | Interface - EBSCOhost Research Databases  Search Screen - Advanced Search  Database - CINAHL | 53,830 |
| S77 | (MH "Self-Management") | Expanders - Apply related words; Apply equivalent subjects  Search modes - Proximity | Interface - EBSCOhost Research Databases  Search Screen - Advanced Search  Database - CINAHL | 5,173 |
| S76 | (MH "Self Care") | Expanders - Apply related words; Apply equivalent subjects  Search modes - Proximity | Interface - EBSCOhost Research Databases  Search Screen - Advanced Search  Database - CINAHL | 47,247 |
| S75 | S44 AND S74 | Expanders - Apply related words; Apply equivalent subjects  Search modes - Proximity | Interface - EBSCOhost Research Databases  Search Screen - Advanced Search  Database - CINAHL | 7 |
| S74 | S55 OR S56 OR S57 OR S58 OR S59 OR S60 OR S61 OR S62 OR S63 OR S64 OR S65 OR S66 OR S67 OR S68 OR S69 OR S70 OR S71 OR S72 OR S73 | Expanders - Apply related words; Apply equivalent subjects  Search modes - Proximity | Interface - EBSCOhost Research Databases  Search Screen - Advanced Search  Database - CINAHL | 189,903 |
| S73 | XB ((care or healthcare or nurs* or program* or rehab* or service#) N3 (digital* or mobile or remote* or tele* or virtual*)) | Expanders - Apply related words; Apply equivalent subjects  Search modes - Proximity | Interface - EBSCOhost Research Databases  Search Screen - Advanced Search  Database - CINAHL | 30,246 |
| S72 | XB ((e W0 rehab*) or (remote W0 rehab*) or tele-rehab* or telerehab* or (virtual W0 rehab*)) | Expanders - Apply related words; Apply equivalent subjects  Search modes - Proximity | Interface - EBSCOhost Research Databases  Search Screen - Advanced Search  Database - CINAHL | 1,513 |
| S71 | (MH "Telerehabilitation") | Expanders - Apply related words; Apply equivalent subjects  Search modes - Proximity | Interface - EBSCOhost Research Databases  Search Screen - Advanced Search  Database - CINAHL | 1,330 |
| S70 | XB ((e W0 nurs*) or enurs* or (remote W0 nurs*) or (tele W0 nurs*) or telenurs* or (virtual W0 nurs*)) | Expanders - Apply related words; Apply equivalent subjects  Search modes - Proximity | Interface - EBSCOhost Research Databases  Search Screen - Advanced Search  Database - CINAHL | 979 |
| S69 | (MH "Telenursing") | Expanders - Apply related words; Apply equivalent subjects  Search modes - Proximity | Interface - EBSCOhost Research Databases  Search Screen - Advanced Search  Database - CINAHL | 2,609 |
| S68 | XB ("e-health" or ehealth or (e W0 med*) or emed* or (remote W0 med*) or "tele care" or telecare or tele-med* or telemed* or "virtual care" or (virtual W0 med*)) | Expanders - Apply related words; Apply equivalent subjects  Search modes - Proximity | Interface - EBSCOhost Research Databases  Search Screen - Advanced Search  Database - CINAHL | 17,740 |
| S67 | (MH "Telehealth") OR (MH "Telemedicine") | Expanders - Apply related words; Apply equivalent subjects  Search modes - Proximity | Interface - EBSCOhost Research Databases  Search Screen - Advanced Search  Database - CINAHL | 34,360 |
| S66 | XB (((computer* or internet* or i-phon* or iphon* or mobile or online or portable or (smart W0 phone*) or smartphone* or software or web) N5 (app or apps or application# or rehab*))) | Expanders - Apply related words; Apply equivalent subjects  Search modes - Proximity | Interface - EBSCOhost Research Databases  Search Screen - Advanced Search  Database - CINAHL | 17,161 |
| S65 | XB ("app-based" or "application-based") | Expanders - Apply related words; Apply equivalent subjects  Search modes - Proximity | Interface - EBSCOhost Research Databases  Search Screen - Advanced Search  Database - CINAHL | 1,094 |
| S64 | (MH "Mobile Applications") | Expanders - Apply related words; Apply equivalent subjects  Search modes - Proximity | Interface - EBSCOhost Research Databases  Search Screen - Advanced Search  Database - CINAHL | 15,342 |
| S63 | XB ("smart phone" or "smart phones" or smartphone#) | Expanders - Apply related words; Apply equivalent subjects  Search modes - Proximity | Interface - EBSCOhost Research Databases  Search Screen - Advanced Search  Database - CINAHL | 10,229 |
| S62 | (MH "Smartphone") | Expanders - Apply related words; Apply equivalent subjects  Search modes - Proximity | Interface - EBSCOhost Research Databases  Search Screen - Advanced Search  Database - CINAHL | 5,496 |
| S61 | XB ((computer-based or Internet-based or ((computer* or Internet*) N3 (care or healthcare or nurs* or program* or rehab* or service#)))) | Expanders - Apply related words; Apply equivalent subjects  Search modes - Proximity | Interface - EBSCOhost Research Databases  Search Screen - Advanced Search  Database - CINAHL | 18,478 |
| S60 | TI (computer* or Internet) | Expanders - Apply related words; Apply equivalent subjects  Search modes - Proximity | Interface - EBSCOhost Research Databases  Search Screen - Advanced Search  Database - CINAHL | 36,567 |
| S59 | (MH "Therapy, Computer Assisted") | Expanders - Apply related words; Apply equivalent subjects  Search modes - Proximity | Interface - EBSCOhost Research Databases  Search Screen - Advanced Search  Database - CINAHL | 5,381 |
| S58 | (MH "Internet-Based Intervention") | Expanders - Apply related words; Apply equivalent subjects  Search modes - Proximity | Interface - EBSCOhost Research Databases  Search Screen - Advanced Search  Database - CINAHL | 2,004 |
| S57 | (MH "Internet") | Expanders - Apply related words; Apply equivalent subjects  Search modes - Proximity | Interface - EBSCOhost Research Databases  Search Screen - Advanced Search  Database - CINAHL | 55,099 |
| S56 | XB ((digital* or virtual*) N3 (care or deliver* or health or healthcare or nursing or program* or rehab* or servic* or treat*)) | Expanders - Apply related words; Apply equivalent subjects  Search modes - Proximity | Interface - EBSCOhost Research Databases  Search Screen - Advanced Search  Database - CINAHL | 19,582 |
| S55 | (MH "Digital Health") | Expanders - Apply related words; Apply equivalent subjects  Search modes - Proximity | Interface - EBSCOhost Research Databases  Search Screen - Advanced Search  Database - CINAHL | 3,159 |
| S54 | S44 AND S53 | Expanders - Apply related words; Apply equivalent subjects  Search modes - Proximity | Interface - EBSCOhost Research Databases  Search Screen - Advanced Search  Database - CINAHL | 42 |
| S53 | S45 OR S46 OR S47 OR S48 OR S49 OR S50 OR S51 OR S52 | Expanders - Apply related words; Apply equivalent subjects  Search modes - Proximity | Interface - EBSCOhost Research Databases  Search Screen - Advanced Search  Database - CINAHL | 198,244 |
| S52 | XB HBCR | Expanders - Apply related words; Apply equivalent subjects  Search modes - Proximity | Interface - EBSCOhost Research Databases  Search Screen - Advanced Search  Database - CINAHL | 24 |
| S51 | XB (((blend* or combo or combination* or hybrid*) N3 (care or "health care" or healthcare or nursing or recover* or rehab* or servic* or support* or treat*))) | Expanders - Apply related words; Apply equivalent subjects  Search modes - Proximity | Interface - EBSCOhost Research Databases  Search Screen - Advanced Search  Database - CINAHL | 16,368 |
| S50 | XB (("at home" or "in home")) | Expanders - Apply related words; Apply equivalent subjects  Search modes - Proximity | Interface - EBSCOhost Research Databases  Search Screen - Advanced Search  Database - CINAHL | 160,661 |
| S49 | XB home W0 setting# | Expanders - Apply related words; Apply equivalent subjects  Search modes - Proximity | Interface - EBSCOhost Research Databases  Search Screen - Advanced Search  Database - CINAHL | 2,815 |
| S48 | XB (((care or "health care" or healthcare or nursing or recover* or rehab* or servic* or support* or treat*) N3 (domestic or domicil* or home#))) | Expanders - Apply related words; Apply equivalent subjects  Search modes - Proximity | Interface - EBSCOhost Research Databases  Search Screen - Advanced Search  Database - CINAHL | 73,991 |
| S47 | XB (("home-based" or homebased or "home care" or homecare or "home health care" or "home healthcare" or ("home health" W0 nurs*) or (home W0 support*) or (home W0 treat*))) | Expanders - Apply related words; Apply equivalent subjects  Search modes - Proximity | Interface - EBSCOhost Research Databases  Search Screen - Advanced Search  Database - CINAHL | 32,729 |
| S46 | (MH "Home Rehabilitation+") | Expanders - Apply related words; Apply equivalent subjects  Search modes - Proximity | Interface - EBSCOhost Research Databases  Search Screen - Advanced Search  Database - CINAHL | 2,792 |
| S45 | (MH "Home Health Care") | Expanders - Apply related words; Apply equivalent subjects  Search modes - Proximity | Interface - EBSCOhost Research Databases  Search Screen - Advanced Search  Database - CINAHL | 27,590 |
| S44 | S32 OR S43 | Expanders - Apply related words; Apply equivalent subjects  Search modes - Proximity | Interface - EBSCOhost Research Databases  Search Screen - Advanced Search  Database - CINAHL | 700 |
| S43 | S33 OR S34 OR S35 OR S36 OR S37 OR S38 OR S39 OR S40 OR S41 OR S42 | Expanders - Apply related words; Apply equivalent subjects  Search modes - Proximity | Interface - EBSCOhost Research Databases  Search Screen - Advanced Search  Database - CINAHL | 165 |
| S42 | XB ((TTVI or TTVR or TTVI-VIV or TTVI-VinV or TTVR-VIV or TTVR-VinV or VinV-TTVI or VinV-TTVR or ViV-TTVI or ViV-TTVR) N3 (recover* or rehab*)) | Expanders - Apply related words; Apply equivalent subjects  Search modes - Proximity | Interface - EBSCOhost Research Databases  Search Screen - Advanced Search  Database - CINAHL | 2 |
| S41 | XB ((TPVI or TPVR or TPVI-VIV or TPVI-VinV or TPVR-VIV or TPVR-VinV or VinV-TPVI or VinV-TPVR or ViV-TPVI or ViV-TPVR) N3 (recover* or rehab*)) | Expanders - Apply related words; Apply equivalent subjects  Search modes - Proximity | Interface - EBSCOhost Research Databases  Search Screen - Advanced Search  Database - CINAHL | 0 |
| S40 | XB ((TMVI or TMVR or TMVI-VIV or TMVI-VinV or TMVR-VIV or TMVR-VinV or VinV-TMVI or VinV-TMVR or ViV-TMVI or ViV-TMVR) N3 (recover* or rehab*)) | Expanders - Apply related words; Apply equivalent subjects  Search modes - Proximity | Interface - EBSCOhost Research Databases  Search Screen - Advanced Search  Database - CINAHL | 4 |
| S39 | XB ((TAVI or TAVR or TAVI-VIV or TAVI-VinV or TAVR-VIV or TAVR-VinV or VinV-TAVI or VinV-TAVR or ViV-TAVI or ViV-TAVR) N3 (recover* or rehab*)) | Expanders - Apply related words; Apply equivalent subjects  Search modes - Proximity | Interface - EBSCOhost Research Databases  Search Screen - Advanced Search  Database - CINAHL | 38 |
| S38 | XB ((STVI or STVR or STVI-VIV or STVI-VinV or STVR-VIV or STVR-VinV or VinV-STVI or VinV-STVR or ViV-STVI or ViV-STVR) N3 (recover* or rehab*)) | Expanders - Apply related words; Apply equivalent subjects  Search modes - Proximity | Interface - EBSCOhost Research Databases  Search Screen - Advanced Search  Database - CINAHL | 1 |
| S37 | XB ((SPVI or SPVR or SPVI-VIV or SPVI-VinV or SPVR-VIV or SPVR-VinV or VinV-SPVI or VinV-SPVR or ViV-SPVI or ViV-SPVR) N3 (recover* or rehab*)) | Expanders - Apply related words; Apply equivalent subjects  Search modes - Proximity | Interface - EBSCOhost Research Databases  Search Screen - Advanced Search  Database - CINAHL | 1 |
| S36 | XB ((SMVI or SMVR or SMVI-VIV or SMVI-VinV or SMVR-VIV or SMVR-VinV or VinV-SMVI or VinV-SMVR or ViV-SMVI or ViV-SMVR) N3 (recover* or rehab*)) | Expanders - Apply related words; Apply equivalent subjects  Search modes - Proximity | Interface - EBSCOhost Research Databases  Search Screen - Advanced Search  Database - CINAHL | 1 |
| S35 | XB ((SAVI or SAVR or SAVI-VIV or SAVI-VinV or SAVR-VIV or SAVR-VinV or VinV-SAVI or VinV-SAVR or ViV-SAVI or ViV-SAVR) N3 (recover* or rehab*)) | Expanders - Apply related words; Apply equivalent subjects  Search modes - Proximity | Interface - EBSCOhost Research Databases  Search Screen - Advanced Search  Database - CINAHL | 5 |
| S34 | XB ((AVI or CVI or HVI or MVI or PVI or TVI or AVR or CVR or HVR or MVR or PVR or TVR) N3 (recover* or rehab*)) | Expanders - Apply related words; Apply equivalent subjects  Search modes - Proximity | Interface - EBSCOhost Research Databases  Search Screen - Advanced Search  Database - CINAHL | 45 |
| S33 | XB (((valve# or valvular) N5 (implant* or prosthes?s* or prosthetic* or reconstruct* or repair* or replac* or surg* or intervent* or operat* or procedur*) N3 (recover* or rehab*))) | Expanders - Apply related words; Apply equivalent subjects  Search modes - Proximity | Interface - EBSCOhost Research Databases  Search Screen - Advanced Search  Database - CINAHL | 87 |
| S32 | S22 AND S31 | Expanders - Apply related words; Apply equivalent subjects  Search modes - Proximity | Interface - EBSCOhost Research Databases  Search Screen - Advanced Search  Database - CINAHL | 607 |
| S31 | S23 OR S24 OR S25 OR S26 OR S27 OR S28 OR S29 OR S30 | Expanders - Apply related words; Apply equivalent subjects  Search modes - Proximity | Interface - EBSCOhost Research Databases  Search Screen - Advanced Search  Database - CINAHL | 68,162 |
| S30 | XB recover* N3 function* | Expanders - Apply related words; Apply equivalent subjects  Search modes - Proximity | Interface - EBSCOhost Research Databases  Search Screen - Advanced Search  Database - CINAHL | 13,051 |
| S29 | (MH "Recovery+") | Expanders - Apply related words; Apply equivalent subjects  Search modes - Proximity | Interface - EBSCOhost Research Databases  Search Screen - Advanced Search  Database - CINAHL | 44,018 |
| S28 | XB ((post-annuloplast* or post-annulo-plast* or (post-annul* W0 reduc*) or (post-annul* W0 repair*) or (post-annul* W0 shorten*) or postannuloplast* or postannulo-plast* or (postannul* W0 reduc*) or (postannul* W0 repair*) or (postannul* W0 shorten*)) N3 (recover* or rehab*)) | Expanders - Apply related words; Apply equivalent subjects  Search modes - Proximity | Interface - EBSCOhost Research Databases  Search Screen - Advanced Search  Database - CINAHL | 1 |
| S27 | XB ((post-implant* or postimplant* or post-operat* or postoperat* or post-procedur* or postprocedur* or post-reconstruct* or postreconstruct* or post-repair* or postrepair* or post-replac* or postreplac* or post-surg* or postsurg*) N3 (recover* or rehab*)) | Expanders - Apply related words; Apply equivalent subjects  Search modes - Proximity | Interface - EBSCOhost Research Databases  Search Screen - Advanced Search  Database - CINAHL | 7,279 |
| S26 | XB ((cardiac or cardio* or CVD or heart*) N3 (recover* or rehab*)) | Expanders - Apply related words; Apply equivalent subjects  Search modes - Proximity | Interface - EBSCOhost Research Databases  Search Screen - Advanced Search  Database - CINAHL | 8,719 |
| S25 | (MH "Rehabilitation, Cardiac+") | Expanders - Apply related words; Apply equivalent subjects  Search modes - Proximity | Interface - EBSCOhost Research Databases  Search Screen - Advanced Search  Database - CINAHL | 5,473 |
| S24 | (MH "Heart Valve Prosthesis+/RH") | Expanders - Apply related words; Apply equivalent subjects  Search modes - Proximity | Interface - EBSCOhost Research Databases  Search Screen - Advanced Search  Database - CINAHL | 31 |
| S23 | (MH "Heart Valve Diseases/RH") | Expanders - Apply related words; Apply equivalent subjects  Search modes - Proximity | Interface - EBSCOhost Research Databases  Search Screen - Advanced Search  Database - CINAHL | 16 |
| S22 | S1 OR S2 OR S3 OR S4 OR S5 OR S6 OR S7 OR S8 OR S9 OR S10 OR S11 OR S12 OR S13 OR S14 OR S15 OR S16 OR S17 OR S18 OR S19 OR S20 OR S21 | Expanders - Apply related words; Apply equivalent subjects  Search modes - Proximity | Interface - EBSCOhost Research Databases  Search Screen - Advanced Search  Database - CINAHL | 31,168 |
| S21 | XB (((aorta# or aortic or cardiac or cardio* or heart# or mitral or pulmonar* or tricuspid or tri-cuspid) N2 (valve# or valvular) N5 (surg* or intervent* or operat* or procedur* or trans-catheter* or transcatheter*))) | Expanders - Apply related words; Apply equivalent subjects  Search modes - Proximity | Interface - EBSCOhost Research Databases  Search Screen - Advanced Search  Database - CINAHL | 11,987 |
| S20 | XB ((aorta# or aortic or mitral or pulmonar* or tricuspid* or tri-cuspid*) W0 (incompeten* or insufficien* or prolaps* or regurgitat*)) | Expanders - Apply related words; Apply equivalent subjects  Search modes - Proximity | Interface - EBSCOhost Research Databases  Search Screen - Advanced Search  Database - CINAHL | 8,666 |
| S19 | XB (((aorta# or aortic or cardiac or cardio* or heart# or mitral or pulmonar* or tricuspid* or tri-cuspid*) N2 (valve# or valvular) N3 (atresia* or incompeten* or insufficien* or prolaps* or regurg* or stenos* or stenotic))) | Expanders - Apply related words; Apply equivalent subjects  Search modes - Proximity | Interface - EBSCOhost Research Databases  Search Screen - Advanced Search  Database - CINAHL | 4,921 |
| S18 | XB (leaflet W0 (reconstruct* or repair* or replac*)) | Expanders - Apply related words; Apply equivalent subjects  Search modes - Proximity | Interface - EBSCOhost Research Databases  Search Screen - Advanced Search  Database - CINAHL | 46 |
| S17 | XB (chord* W0 (reconstruct* or repair* or replac*)) | Expanders - Apply related words; Apply equivalent subjects  Search modes - Proximity | Interface - EBSCOhost Research Databases  Search Screen - Advanced Search  Database - CINAHL | 20 |
| S16 | XB (PTAA or PTMA or PTPA or PTTA) | Expanders - Apply related words; Apply equivalent subjects  Search modes - Proximity | Interface - EBSCOhost Research Databases  Search Screen - Advanced Search  Database - CINAHL | 25 |
| S15 | XB (annuloplast* or (annulo W0 plast*) or (annul* W0 reduc*) or (annul* W0 repair*) or (annul* W0 shorten*)) | Expanders - Apply related words; Apply equivalent subjects  Search modes - Proximity | Interface - EBSCOhost Research Databases  Search Screen - Advanced Search  Database - CINAHL | 676 |
| S14 | (MH "Cardiac Valve Annuloplasty+") | Expanders - Apply related words; Apply equivalent subjects  Search modes - Proximity | Interface - EBSCOhost Research Databases  Search Screen - Advanced Search  Database - CINAHL | 485 |
| S13 | XB (TTVI or TTVR or TTVI-VIV or TTVI-VinV or TTVR-VIV or TTVR-VinV or VinV-TTVI or VinV-TTVR or ViV-TTVI or ViV-TTVR) | Expanders - Apply related words; Apply equivalent subjects  Search modes - Proximity | Interface - EBSCOhost Research Databases  Search Screen - Advanced Search  Database - CINAHL | 24 |
| S12 | XB (TPVI or TPVR or TPVI-VIV or TPVI-VinV or TPVR-VIV or TPVR-VinV or VinV-TPVI or VinV-TPVR or ViV-TPVI or ViV-TPVR) | Expanders - Apply related words; Apply equivalent subjects  Search modes - Proximity | Interface - EBSCOhost Research Databases  Search Screen - Advanced Search  Database - CINAHL | 32 |
| S11 | XB (TMVI or TMVR or TMVI-VIV or TMVI-VinV or TMVR-VIV or TMVR-VinV or VinV-TMVI or VinV-TMVR or ViV-TMVI or ViV-TMVR) | Expanders - Apply related words; Apply equivalent subjects  Search modes - Proximity | Interface - EBSCOhost Research Databases  Search Screen - Advanced Search  Database - CINAHL | 150 |
| S10 | XB (TAVI or TAVR or TAVI-VIV or TAVI-VinV or TAVR-VIV or TAVR-VinV or VinV-TAVI or VinV-TAVR or ViV-TAVI or ViV-TAVR) | Expanders - Apply related words; Apply equivalent subjects  Search modes - Proximity | Interface - EBSCOhost Research Databases  Search Screen - Advanced Search  Database - CINAHL | 3,883 |
| S9 | XB (STVI or STVR or STVI-VIV or STVI-VinV or STVR-VIV or STVR-VinV or VinV-STVI or VinV-STVR or ViV-STVI or ViV-STVR) | Expanders - Apply related words; Apply equivalent subjects  Search modes - Proximity | Interface - EBSCOhost Research Databases  Search Screen - Advanced Search  Database - CINAHL | 3 |
| S8 | XB (SPVI or SPVR or SPVI-VIV or SPVI-VinV or SPVR-VIV or SPVR-VinV or VinV-SPVI or VinV-SPVR or ViV-SPVI or ViV-SPVR) | Expanders - Apply related words; Apply equivalent subjects  Search modes - Proximity | Interface - EBSCOhost Research Databases  Search Screen - Advanced Search  Database - CINAHL | 11 |
| S7 | XB (SMVI or SMVR or SMVI-VIV or SMVI-VinV or SMVR-VIV or SMVR-VinV or VinV-SMVI or VinV-SMVR or ViV-SMVI or ViV-SMVR) | Expanders - Apply related words; Apply equivalent subjects  Search modes - Proximity | Interface - EBSCOhost Research Databases  Search Screen - Advanced Search  Database - CINAHL | 13 |
| S6 | XB (SAVI or SAVR or SAVI-VIV or SAVI-VinV or SAVR-VIV or SAVR-VinV or VinV-SAVI or VinV-SAVR or ViV-SAVI or ViV-SAVR) | Expanders - Apply related words; Apply equivalent subjects  Search modes - Proximity | Interface - EBSCOhost Research Databases  Search Screen - Advanced Search  Database - CINAHL | 523 |
| S5 | XB ((valve# or valvular) AND (AVI or CVI or HVI or MVI or PVI or TVI or AVR or CVR or HVR or MVR or PVR or TVR)) | Expanders - Apply related words; Apply equivalent subjects  Search modes - Proximity | Interface - EBSCOhost Research Databases  Search Screen - Advanced Search  Database - CINAHL | 1,103 |
| S4 | XB ((valve# or valvular) N5 (implant* or reconstruct* or repair* or replac*)) | Expanders - Apply related words; Apply equivalent subjects  Search modes - Proximity | Interface - EBSCOhost Research Databases  Search Screen - Advanced Search  Database - CINAHL | 15,262 |
| S3 | XB (valvo-plast* or valvoplast* or valvo-tom* or valvotom* or valvulo-plast* or valvuloplast* or valvulo-tom* or valvulotom*) | Expanders - Apply related words; Apply equivalent subjects  Search modes - Proximity | Interface - EBSCOhost Research Databases  Search Screen - Advanced Search  Database - CINAHL | 1,091 |
| S2 | (MH "Heart Valve Prosthesis+") | Expanders - Apply related words; Apply equivalent subjects  Search modes - Proximity | Interface - EBSCOhost Research Databases  Search Screen - Advanced Search  Database - CINAHL | 9,384 |
| S1 | (MH "Heart Valve Diseases/SU") | Expanders - Apply related words; Apply equivalent subjects  Search modes - Proximity | Interface - EBSCOhost Research Databases  Search Screen - Advanced Search  Database - CINAHL | 2,040 |

Web of Science (Core Databases)

| Set # | Search Query | Results |
| --- | --- | --- |
| 1 | valvo-plast* or valvoplast* or valvo-tom* or valvotom* or valvulo-plast* or valvuloplast* or valvulo-tom* or valvulotom* (Topic) OR (valve or valves or valvular) NEAR/5 (implant* or reconstruct* or repair* or replac*) (Topic) OR (valve or valves or valvular) NEAR/5 (AVI or CVI or HVI or MVI or PVI or TVI or AVR or CVR or HVR or MVR or PVR or TVR) (Topic) | 91602 |
| 2 | SAVI or SAVR or SAVI-VIV or SAVI-VinV or SAVR-VIV or SAVR-VinV or VinV-SAVI or VinV-SAVR or ViV-SAVI or ViV-SAVR (Topic) OR SMVI or SMVR or SMVI-VIV or SMVI-VinV or SMVR-VIV or SMVR-VinV or VinV-SMVI or VinV-SMVR or ViV-SMVI or ViV-SMVR (Topic) OR SPVI or SPVR or SPVI-VIV or SPVI-VinV or SPVR-VIV or SPVR-VinV or VinV-SPVI or VinV-SPVR or ViV-SPVI or ViV-SPVR (Topic) OR STVI or STVR or STVI-VIV or STVI-VinV or STVR-VIV or STVR-VinV or VinV-STVI or VinV-STVR or ViV-STVI or ViV-STVR (Topic) OR TAVI or TAVR or TAVI-VIV or TAVI-VinV or TAVR-VIV or TAVR-VinV or VinV-TAVI or VinV-TAVR or ViV-TAVI or ViV-TAVR (Topic) OR TMVI or TMVR or TMVI-VIV or TMVI-VinV or TMVR-VIV or TMVR-VinV or VinV-TMVI or VinV-TMVR or ViV-TMVI or ViV-TMVR (Topic) OR TPVI or TPVR or TPVI-VIV or TPVI-VinV or TPVR-VIV or TPVR-VinV or VinV-TPVI or VinV-TPVR or ViV-TPVI or ViV-TPVR (Topic) OR TTVI or TTVR or TTVI-VIV or TTVI-VinV or TTVR-VIV or TTVR-VinV or VinV-TTVI or VinV-TTVR or ViV-TTVI or ViV-TTVR (Topic) | 21600 |
| 3 | annuloplast* or annulo-plast* or (annul* NEAR/0 reduc*) or (annul* NEAR/0 repair*) or (annul* NEAR/0 shorten*) (Topic) OR PTAA or PTMA or PTPA or PTTA (Topic) OR chord* NEAR/0 (reconstruct* or repair* or replac*) (Topic) OR leaflet NEAR/0 (reconstruct* or repair* or replac*) (Topic) | 8542 |
| 4 | ((aorta or aortas or aortic or cardiac or cardio* or heart or hearts or mitral or pulmonar* or tricuspid* or tri-cuspid*) NEAR/2 (valve or valves or valvular) NEAR/3 (atresia* or incompeten* or insufficien* or prolaps* or regurg* or stenos* or stenotic)) (Topic) OR (aorta or aortas or aortic or mitral or pulmonar* or tricuspid* or tri-cuspid*) NEAR/0 (incompeten* or insufficien* or prolaps* or regurgitat*) (Topic) OR ((aorta or aortas or aortas or aortic or mitral or pulmonar* or tricuspid* or tri-cuspid*) NEAR/2 (valve or valves or valvular) NEAR/5 (surg* or intervent* or operat* or procedur* or trans-catheter* or transcatheter*)) (Topic) | 105815 |
| 5 | #1 OR #2 OR #3 OR #4 | 151893 |
| 6 | (cardiac or cardio* or CVD or heart*) NEAR/3 (recover* or rehab*) (Topic) OR (post-implant* or postimplant* or post-operat* or postoperat* or post-procedur* or postprocedur* or post-reconstruct* or postreconstruct* or post-repair* or postrepair* or post-replac* or postreplac* or post-surg* or postsurg*) NEAR/3 (recover* or rehab*) (Topic) OR (post-annuloplast* or post-annulo-plast* or (post-annul* NEAR/0 reduc*) or (post-annul* NEAR/0 repair*) or (post-annul* NEAR/0 shorten*) or postannuloplast* or postannulo-plast* or (postannul* NEAR/0 reduc*) or (postannul* NEAR/0 repair*) or (postannul* NEAR/0 shorten*)) NEAR/3 (recover* or rehab*) (Topic) | 58618 |
| 7 | recover* NEAR/3 function* (Topic) | 87323 |
| 8 | #6 OR #7 | 139437 |
| 9 | ((valve or valves or valvular) NEAR/5 (implant* or prostheses* or prosthesis* or prosthetic* or reconstruct* or repair* or replac* or surg* or intervent* or operat* or procedur*) NEAR/3 (recover* or rehab*)) (Topic) OR (AVI or CVI or HVI or MVI or PVI or TVI or AVR or CVR or HVR or MVR or PVR or TVR) NEAR/3 (recover* or rehab*) (Topic) OR (SAVI or SAVR or SAVI-VIV or SAVI-VinV or SAVR-VIV or SAVR-VinV or VinV-SAVI or VinV-SAVR or ViV-SAVI or ViV-SAVR) NEAR/3 (recover* or rehab*) (Topic) OR (SMVI or SMVR or SMVI-VIV or SMVI-VinV or SMVR-VIV or SMVR-VinV or VinV-SMVI or VinV-SMVR or ViV-SMVI or ViV-SMVR) NEAR/3 (recover* or rehab*) (Topic) OR (SPVI or SPVR or SPVI-VIV or SPVI-VinV or SPVR-VIV or SPVR-VinV or VinV-SPVI or VinV-SPVR or ViV-SPVI or ViV-SPVR) NEAR/3 (recover* or rehab*) (Topic) OR (STVI or STVR or STVI-VIV or STVI-VinV or STVR-VIV or STVR-VinV or VinV-STVI or VinV-STVR or ViV-STVI or ViV-STVR) NEAR/3 (recover* or rehab*) (Topic) | 638 |
| 10 | (TAVI or TAVR or TAVI-VIV or TAVI-VinV or TAVR-VIV or TAVR-VinV or VinV-TAVI or VinV-TAVR or ViV-TAVI or ViV-TAVR) NEAR/3 (recover* or rehab*) (Topic) OR (TMVI or TMVR or TMVI-VIV or TMVI-VinV or TMVR-VIV or TMVR-VinV or VinV-TMVI or VinV-TMVR or ViV-TMVI or ViV-TMVR) NEAR/3 (recover* or rehab*) (Topic) OR (TPVI or TPVR or TPVI-VIV or TPVI-VinV or TPVR-VIV or TPVR-VinV or VinV-TPVI or VinV-TPVR or ViV-TPVI or ViV-TPVR) NEAR/3 (recover* or rehab*) (Topic) OR (TTVI or TTVR or TTVI-VIV or TTVI-VinV or TTVR-VIV or TTVR-VinV or VinV-TTVI or VinV-TTVR or ViV-TTVI or ViV-TTVR) NEAR/3 (recover* or rehab*) (Topic) | 123 |
| 11 | #9 OR #10 | 739 |
| 12 | #5 AND #8 | 1435 |
| 13 | #11 OR #12 | 1978 |
| 14 | home-based or homebased or "home care" or homecare or "home health care" or "home healthcare" or ("home health" NEAR/0 nurs*) or (home NEAR/0 support*) or (home NEAR/0 treat*) (Topic) OR (care or "health care" or healthcare or nursing or recover* or rehab* or servic* or support* or treat*) NEAR/3 (domestic or domicil* or home or homes) (Topic) OR "home setting" or "home settings" (Topic) OR "at home" or "in home" (Topic) OR (blend* or combo or combination* or hybrid*) NEAR/3 (care or "health care" or healthcare or nursing or recover* or rehab* or servic* or support* or treat*) (Topic) OR HBCR (Topic) | 400345 |
| 15 | #13 AND #14 | 55 |
| 16 | (digital* or virtual*) NEAR/3 (care or deliver* or health or healthcare or nursing or program* or rehab* or servic* or treat*) (Topic) OR computer* or Internet (Title) OR computer-based or Internet-based or ((computer* or Internet*) NEAR/3 (care or healthcare or nurs* or program* or rehab* or service or services)) (Topic) OR "smart phone" or "smart phones" or smartphone* (Topic) OR "app-based" or "application-based" (Topic) OR (computer* or internet* or "i-phone" or "i-phones" or iphon* or mobile or online or portable or (smart NEAR/0 phone*) or smartphone* or software or web) NEAR/5 (app or apps or application* or rehab*) (Topic) | 884831 |
| 17 | "e-health" or ehealth or (e NEAR/0 med*) or emed* or (remote NEAR/0 med*) or "tele care" or telecare or tele-med* or telemed* or "virtual care" or (virtual NEAR/0 med*) (Topic) OR (e NEAR/0 nurs*) or enurs* or (remote NEAR/0 nurs*) or (tele NEAR/0 nurs*) or telenurs* or (virtual NEAR/0 nurs*) (Topic) OR (e NEAR/0 rehab*) or (remote NEAR/0 rehab*) or (tele NEAR/0 rehab*) or telerehab* or (virtual NEAR/0 rehab*) (Topic) OR (care or healthcare or nurs* or program* or rehab* or service or services) NEAR/3 (digital* or mobile or remote* or tele* or virtual*) (Topic) | 202783 |
| 18 | #17 OR #16 | 1012078 |
| 19 | #18 AND #13 | 27 |
| 20 | (personal or self) NEAR/3 (care or manag*) (Topic) | 138329 |
| 21 | #20 AND #13 | 12 |
| 22 | #21 OR #19 OR #15 | 76 |

Clinical Trials. gov

("cardiac rehabilitation" OR HBCR OR "home rehabilitation" OR telerehabilitation OR tele-rehabilitation OR telerehab OR "digital rehabilitation" OR "remote rehabilitation")

AND

("valve replacement" OR "valve repair" OR TAVR OR TAVI OR SAVR OR " heart valve surgery")

Total number of results: 51
