# Supplementary material for: Effectiveness, Efficacy, and Safety of Home-Based Cardiac Rehabilitation Following Valve Replacement or Repair: Protocol for a Systematic Review
Source: JMIR Res Protoc. 2026 Aug 6;15:e86832. doi: 10.2196/86832 (PMC13494523; doi:10.2196/86832)
Supplement: Supplementary file 4 [file resprot_v15i1e86832_app4.docx]

| Intervention/Comparator elements | Description of the elements |
| --- | --- |
| Phase of the CR program | a. The specific cardiac rehabilitation (CR) phase being delivered (e.g., phase I, phase II). b. How many days post-surgery, or post-hospital discharge did the intervention/CR program start? |
| Setting | a. The location where the participants receive the intervention (e.g., home, nursing care homes, hospital). |
| Provider(s)/facilitator of the Program | a. Who delivers the interventions (e.g., doctor, nurse)? b. What are their roles? |
| Training provided to Provider/Facilitator | a. Any training or preparation received by the providers to deliver the intervention (e.g., study-specific protocol training, formal certification)? b. How did they receive the training (e.g., real-time training sessions, training material)? |
| Theory/Rationale/Goal Behind the Intervention | a. The theoretical framework or rationale used to design the intervention (e.g., to increase program adherence in CR). b. What is the goal(s) of the elements of the intervention? |
| Training Provided to Participants | a. Any orientation or preparatory training provided to participants (e.g., Instructions on equipment use, safety education)? b. How did they receive the training (e.g., real-time training sessions, training material)? |
| Core Components of the Intervention/Program | a. Key components included in the intervention (e.g., exercise training, counselling, education). b. Details on the exercise training (e.g., type of exercises, intensity). c. Details on other components included in the intervention. |
| Tailoring of the Intervention/Program | a. Was the intervention personalized to participant needs and their baseline assessment (e.g., individualized exercise plans and counselling)?  - If personalized at baseline, is it also adjusted through the intervention period? - Why, how often, and how?  b. If the intervention was not personalized at the individual level, was it designed to be progressively adapted during the intervention period in a standardized way for all participants (e.g., a structured protocol involving gradual increases in exercise intensity, duration, or complexity over time)?  - Why, when, and how? |
| Modifications to the Intervention/Program | a. Any changes made to the intervention protocol during the study period (e.g., adjustments, content revisions, COVID-19 adaptations)?  - Why, when, and how? |
| Modes of Delivery | a. The platform(s) or tool(s) used to deliver the intervention (e.g., mHealth tech, telehealth, telephone, in-person visits). |
| Delivery Format of the Intervention/Program | a. Were the sessions/program components delivered synchronously, asynchronously, or hybrid? |
| Duration of the Intervention/Program | a. Total length of the intervention from start to finish. |
| Frequency and Duration of Each Session | a. Number of sessions per week, or total number of sessions over the entire period of the intervention. This might be exercise sessions, counselling sessions, etc. They will be extracted separately, where possible. b. Average length of each session? |
| Session Format (Group vs Individual) | a. Were the sessions conducted in a group setting, individually, or in a hybrid approach? |
| Monitoring/Supervision/Follow-Up | a. Methods used to monitor participants and provide ongoing supervision or follow-up.  - Who monitors, When, How often were they done?  b. Methods used to monitor the compliance of participants to the intervention.  - Who monitors, When, How often were they done?  c. Was the intervention adherence or fidelity assessed? - What is the intervention adherence or fidelity, if assessed? |
| Other | Any relevant additional details about the intervention not captured in the above elements. |

*Table 3. Data extraction template, guided by the TIDieR framework, for intervention and comparators.*
